# Drosophila Sirtuin 6 mediates developmental diet‐dependent programming of adult physiology and survival

**DOI:** 10.1111/acel.13576

**Published:** 2022-03-02

**Authors:** Namrata Shukla, Ullas Kolthur‐Seetharam

**Affiliations:** ^1^ Department of Biological Sciences Tata Institute of Fundamental Research Mumbai India; ^2^ Tata Institute of Fundamental Research‐Hyderabad (TIFR‐H) Hyderabad India

**Keywords:** ageing, development, ecdysone, healthspan, lifespan, metabolism, mitochondria, oxidative stress, physical activity, *SIRT6*

## Abstract

Organisms in the wild experience unpredictable and diverse food availability throughout their lifespan. Over‐/under‐nutrition during development and in adulthood is known to dictate organismal survival and fitness. Studies using model systems have also established long‐term effects of developmental dietary alterations on life‐history traits. However, the underlining genetic/molecular factors, which differentially couple nutrient inputs during development with fitness later in life are far less understood. Using *Drosophila* and loss/gain of function perturbations, our serendipitous findings demonstrate an essential role of *Sirtuin 6* in regulating larval developmental kinetics, in a nutrient‐dependent manner. The absence of *Sirt6* affected ecdysone and insulin signalling and led to accelerated larval development. Moreover, varying dietary glucose and yeast during larval stages resulted in enhanced susceptibility to metabolic and oxidative stress in adults. We also demonstrate an evolutionarily conserved role for *Sirt6* in regulating physiological homeostasis, physical activity and organismal lifespan, known only in mammals until now. Our results highlight gene‐diet interactions that dictate thresholding of nutrient inputs and physiological plasticity, operative across development and adulthood. In summary, besides showing its role in invertebrate ageing, our study also identifies *Sirt6* as a key factor that programs macronutrient‐dependent life‐history traits.

## Graphical abstract

Larval nutritional inputs dictate adult physiological fitness. Drosophila Sirt6 plays an evolutionarily conserved role in determining organismal physiology and survival. Sirt6 is necessary for physiological plasticity and for regulation of macronutrient‐dependent life‐history traits

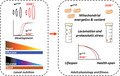

## 1 INTRODUCTION

Seasonal variations in diet availability impact organismal fitness throughout life. Life‐history changes, both during development and in adulthood, cumulatively dictate the ability to mitigate stresses and hence contribute to the survival of individuals in a population/species (Behrman et al., 2015; Brankatschk et al., 2018; Gerofotis et al., 2019). Notably, nutrient availability and diet composition during early development, in coordination with environmental variations, have been shown to be important factors governing adult physiology (Brankatschk et al., 2018; Langley‐Evans, 2015; Palgunow et al., 2012; Rehman & Varghese, 2021). Studies in mammals, including humans, have highlighted the causal role of parental diets and metabolic inputs during development in predisposition to lifestyle and age‐associated MetS (metabolic syndromes) including obesity, type 2 diabetes, hypertension and stroke (Delpierre et al., 2016; Hibshman et al., 2016; Jahan‐Mihan et al., 2015; Parlee & MacDougald, 2014; Watkins & Sinclair, 2014). Therefore, from an interventional perspective, it is important to further elucidate both phenomenological and mechanistic workings of late‐onset diseases, which have origins from differential nutrient inputs during development.

In case of holometabolous insects like *Drosophila*, adult body size and physiology are pre‐determined by the nutritional status during larval development (Güler et al., 2015; Reis, 2016; Shingleton et al., 2008). Several studies have enumerated the contribution of carbohydrates, yeast (protein) and fats in regulating larval development time, by interacting with developmentally important signalling cascades viz. steroid hormones, insulin and TOR pathways (Buhler et al., 2018; Danielsen et al., 2013; Layalle et al., 2008; Liu et al., 2018), and in turn, shaping adult physiology (Reis, 2016). For example, yeast deprivation (considered to cause protein restriction) in larval diet has been shown to moderately increase lifespan, in a developmental stage‐dependent manner (Danielsen et al., 2013; May et al., 2015; Tu & Tatar, 2003).

Studies in past have posited antagonistic and pleiotropic interactions between pathways involved in development and ageing (Blagosklonny, 2010). This notion is supported by findings, which show that while excess nutrient inputs during development favour accelerated growth, overnutrition during adulthood is negatively associated with organismal fitness and survival (Parlee & MacDougald, 2014). Moreover, given that differential macronutrient inputs, which determine developmental kinetics, also impinge on adult physiology, molecular and genetic factors that couple these two remain elusive.

In this regard, it is intuitive to invoke a plausible role for epigenetic regulators, besides others, in coupling developmental nutrient inputs with growth and adult physiology. Nuclear sirtuins, nicotinamide adenine dinucleotide (NAD+) dependent deacylases, are known to directly link metabolic cues to gene expression programs that govern organismal survival (Banerjee et al., 2013, 2017; Houtkooper et al., 2012; Parik et al., 2018). However, a potential role for these metabolic sensors in mediating life‐history changes, emanating from altered developmental nutritional inputs, has not been addressed thus far.

Mammalian studies have established *SIRT6* as an anti‐ageing factor (Chang et al., 2020; Mostoslavsky et al., 2006; Tasselli et al., 2017). This has been largely attributed to *SIRT6* dependent regulation of chromatin and consequently altered gene expression, DNA damage, insulin signalling, and glucose and fat metabolism (Chang et al., 2020; Tasselli et al., 2017). Nevertheless, evolutionary conservation of its ability to dictate physiological homeostasis and ageing, especially in invertebrates, remains unknown. While absence of *SIRT6* has been associated with several developmental and adult disorders/pathologies (Chang et al., 2020; Mostoslavsky et al., 2006; Tasselli et al., 2017), whether or not its functions impinge on coupling developmental nutrition inputs to adult survival is still unexplored.

Here, we describe the importance of nutrient inputs, and their dependence on *Sirt6*, in exerting a control over developmental progression and its correlates with physiological fitness and healthspan in adulthood. Besides demonstrating the evolutionarily conserved role of *Sirt6* in regulating ageing in *Drosophila*, we illustrate its relevance during larval development, which was previously unknown. Importantly, our observations indicate antagonism between accelerated larval development and adult stress resistance, which is exacerbated in the absence of *Sirt6*. Our results postulate developmental hypertrophy as a detrimental factor for adult physiological fitness. Additionally, they also highlight the need to reveal the underlying mechanisms that mediate plasticity/memory of life‐history nutrient changes, especially in the wild.

## 2 RESULTS

### 2.1 Loss of *Sirt6* causes accelerated larval development and adult hypertrophy in *Drosophila*

Aligning mammalian SIRT6 with *Drosophila* SIRT6 showed conservation of the active site residues and the NAD+ binding domain displayed 80% identity with both human and mouse SIRT6 (Figure S1a). To study the function of *Sirt6* in an invertebrate system, we generated two independent CRISPR mutant fly lines (denoted as *Sirt6* ^−/−^ *^bck^* ^−^ *^L1^* and *Sirt6* ^−/−^ *^bck^* ^−^ *^L2^*, *“bck”* for backcrossed to control *w^1118^*), as indicated (Figure S1b,c). The genetic deletion and subsequent loss of *Sirt6* expression were confirmed by genotyping and quantitative RT‐PCR (Figure S1d,e).

**FIGURE 1:**
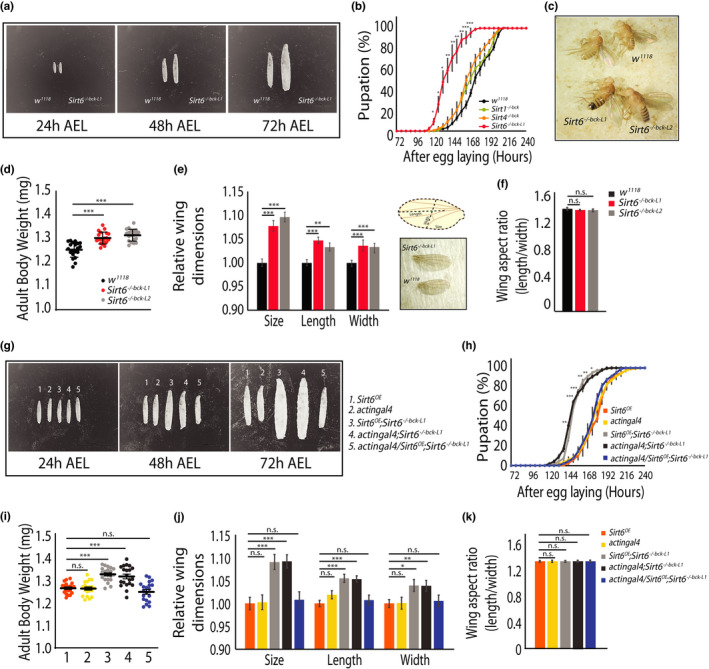
Loss of *Sirt6* causes accelerated larval development and adult hypertrophy in *Drosophila*. (a) Larval body size at 24, 48 and 72 h post‐synchronised egg laying. *w^1118^* (control) and *Sirt6^−/−bck−L1^* larvae were imaged at 12.5× magnification. (b) Percentage pupation in backcrossed *Sirt1^−/−^^bck^*, *Sirt4^−/−^^bck^* 4 and *Sirt6^−/−bck−L1^* larvae compared to *w^1118^* flies (N = 3, n = 150–200). (c) Representative image of 3–5 day old *w^1118^* and *Sirt6^−/−bck−L1/2^* flies. (d) Body weight measurement in *w^1118^* and *Sirt6^−/−bck−L1/2^* flies (N = 3, n = 20–25 per genotype). (e) Relative wing dimensions in *w^1118^* and *Sirt6^−/−bck−L1/2^* flies (N = 3, n = 20–25). (f) Wing aspect ratio in *w^1118^* and *Sirt6^−/−bck−L1/2^* flies, as computed from (e). (g) Larval body size at 24, 48 and 72 h post‐synchronised egg laying in the following genotypes: 1‐*Sirt6^OE^*, 2‐*actingal4*, 3‐*Sirt6^OE^*; *Sirt6^−/−bck−L1^*, 4‐*actingal4*; *Sirt6^−/−bck−L1^* and 5‐*actingal4/Sirt6^OE^*; *Sirt6^−/−bck−L1^*. (h‐k) Genetic rescue of *Sirt6* restores early pupation (H), body weight (I), wing dimensions (J) and wing aspect ratio (k) phenotype (N = 3, n = 20–25 per genotype). All data presented are mean ± SEM Asterisk depicts *p* values (*p < 0.05, **p < 0.01 and ***p < 0.001) as observed by Student's *t*‐test and two‐way ANOVA, as applicable

To our surprise, *Sirt6* mutant flies displayed a developmental phenotype, which has not been reported previously in vertebrates. Larvae lacking *Sirt6* were substantially larger at 72 h after egg laying (AEL) (Figure 1a; Figure S1f). It was also interesting to note that while larval sizes were comparable at 24 h AEL between the control *w^1118^* and *Sirt6* ^−/−^ *^bck^* ^−^ *^L1^* genotypes, increase in size and weight of mutant *Sirt6* larvae became evident at 48h AEL (Figure 1a; Figure S1f). Further, we also found early pupation of these mutant larvae (Figure 1b) and the two independent mutant lines phenocopied each other. This early developmental phenotype was *Sirtuin 6* specific as we did not observe any alterations in developmental progression for other sirtuins that were assessed viz. *Sirt1* ^−/−^ *^bck^* and *Sirt4* ^−/−^ *^bck^*, when compared to control *w^1118^* larvae (Figure 1b).

In addition to accelerated developmental progression, *Sirt6* ^−/−^ *^bck^* ^−^ *^L1^* ^/^ *^2^* mutants displayed a hypertrophy phenotype in the adults, wherein flies lacking *Sirt6* were larger (Figure 1c) and weighed significantly more than the control *w^1118^* flies (Figure 1d). Typically, wing size and aspect ratio have been used as indicators to assess hypertrophy (Guerra et al., 1997). As shown in Figure 1e, we observed a significant increase in the size of the wings in *Sirt6* ^−/−^ *^bck^* ^−^ *^L1^* ^/^ *^2^* flies when compared to *w^1118^* controls. Measuring aspect ratios across genotypes clearly indicated that this was not due to abnormal development along any particular axis (Figure 1f).

In order to confirm *Sirt6* dependence of these phenotypes, we genetically rescued *Sirt6* in mutant flies and also overexpressed it in control wild‐type flies to assess gain of function. To this end, we cloned *Drosophila Sirt6* in a pUAST‐attB plasmid and generated *UAS*‐*Sirt6* (denoted henceforth as *Sirt6^OE^*) transgenic flies (detailed in Methods under 'Generation of transgenic *UAS‐Sirt6 (Sirt6* *^OE^) fly'*). As seen in Figure S1g–l, contrary to loss of function, overexpression of *Sirt6* (*actingal4*/*Sirt6^OE^*) did not have any effect on larval development, pupation or adult fly weight and wing phenotype, compared to control flies. Importantly, genetic rescue of *Sirt6* (*actingal4*/*Sirt6^OE^*; *Sirt6* ^−/−^ *^bck^*) (Figure S1m) restored larval development (Figure 1g,h) as well as adult body weight and wing phenotype, which was comparable to the respective controls (Figure 1i–k). Together, these not only validated *Sirt6* dependence of growth and development but also indicated that these were particularly sensitive to *Sirt6* loss of function.

### 2.2 *Sirt6* absence accelerates larval development with no change in critical weight

In invertebrates, larval development and pupariation are intrinsically dependent upon attainment of critical weight, which acts as a size checkpoint to determine end of larval growth period and beginning of metamorphosis (Moed et al., 1999; Hironaka et al., 2019). Therefore, we scored for critical weight and developmental progression, both morphologically and at a molecular level.

**FIGURE 2:**
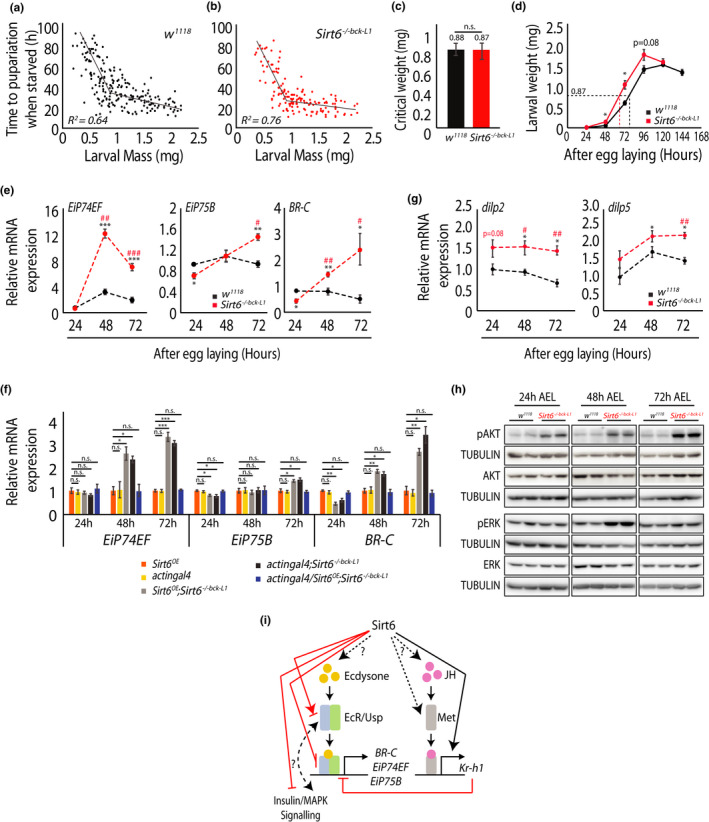
*Sirt6* absence accelerates larval development with no change in critical weight. (a, b) Time to pupariation in *w^1118^* (a) and *Sirt6^−/−bck−L1^* (b) larvae starved at different weights. The break in regression line indicates the time when critical weight has been reached (N = 3, n = 250–300). (c) Critical weight computed from (a, b) for *w^1118^* and *Sirt6^−/−bck−L1^* larvae. (d) Larval weight gain in *w^1118^* and *Sirt6^−/−bck−L1^* post‐synchronised egg laying. Mutant *Sirt6* larvae reach critical weight earlier than controls. (N = 3, n = 20–25 per genotype). (e, f) Relative change in expression of ecdysone target genes in *Sirt6^−/−bck−L1^* larvae compared to *w^1118^* during the course of larval development (e) and their rescue with transgenic *Sirt6* expression (f), as indicted (N = 3, n = 3 with 10–20 larvae per n). Asterisk depicts comparison with *w^1118^* at 24 h and hashtags depict comparison of the *Sirt6^−/−bck−L1^* to *w^1118^*, at the respective time points, as indicated. (g) Relative change in expression of *dilp2* and *dilp5* in *Sirt6^−/−bck−L1^* larvae compared to *w^1118^* during the course of larval development (N = 3, n = 3 with 10–20 larvae per n). Asterisk depicts comparison with *w^1118^* at 24 h and hashtags depict comparison of the *Sirt6^−/−bck−L1^* to *w^1118^*, at the respective time points, as indicated. (H) Representative western blots showing phosphorylation of AKT and ERK at 24, 48 and 72 h post‐egg laying in control *w^1118^* and *Sirt6^−/−bck−L1^* larvae. (i) Schematic depicting *Sirt6*‐mediated regulation of ecdysone and insulin signalling. All data presented are mean ± SEM. Asterisk and hashtags depict *p* values (*, ^#^ *p* < 0.05, **, ^##^ *p* < 0.01 and ***, ^###^ *p* < 0.001) as observed by Student's *t*‐test and two‐way ANOVA, wherever applicable

On assessing time to pupariation (TTP) post‐starvation and subsequent break‐point analysis (Muggeo, 2003; also see Methods sub‐section 'Critical weight estimation'), we found no significant change in the critical weights of control *w^1118^* and *Sirt6* ^−/−^ *^bck^* ^−^ *^L1^* flies (Figure 2a–c). However, *Sirt6* mutant larvae attained critical weight earlier (68 h), when compared to the controls (78 h; Figure 2d). This was interesting since we found that neither *Sirt6* mRNA nor NAD+, its co‐substrate, varied significantly during larval stages (Figure S2a). Developmental kinetics and moulting in flies are under the control of several endocrine and growth signalling cascades, ecdysone being one of the key regulators (Koyama et al., 2020; Liu et al., 2018; Yamanaka et al., 2013). Albeit larval *Sirt6* expression was unaltered (Figure S2a), profiling for expression of ecdysone signalling genes, including targets of ecdysone receptor—*EiP74EF*, *EiP75B* and *BR*‐*C*, showed both temporal and quantitative changes when *Sirt6* was absent (Figure 2e). Specifically, at 72 h post‐egg laying, there was a significant upregulation of the key ecdysone target genes (Figure 2e). This was consistent with increased expression of ecdysone co‐receptor, *Ultraspiracle* (Figure S2b). Importantly, there was a decrease in the juvenile hormone target *Kr*‐*h1* (Figure S2b), which is known to antagonise ecdysone signalling (Yamanaka et al., 2013). These gene expression changes, similar to the larval growth phenotype (Figure 1k), were rescued by re‐introducing *Sirt6* in the mutant background *(actingal4*/*Sirt6^OE^*; *Sirt6* ^−/−^ *^bck^*; Figure 2f).

Given that insulin signalling is an important regulator of early development and its interplay with ecdysone signalling has been shown to regulate developmental progression (Koyama et al., 2020; Liu et al., 2018; Yamanaka et al., 2013), we next scored for expression of drosophila insulin‐like peptides. We found elevated levels of *dilp2* and *dilp5* in the *Sirt6* ^−/−^ *^bck^* larvae, which were rescued by transgenic overexpression of *Sirt6* (Figure 2g; Figure S2c). This was correlated with heightened insulin/growth‐factor signalling, as evidenced by enhanced phosphorylation of AKT and ERK (Figure 2h). Taken together with earlier reports (Sundaresan et al., 2012), this also illustrated evolutionary conserved role for *Sirt6* in regulating insulin signalling.

The results described above clearly demonstrate that *Sirt6* exerts control over both steroid hormone and endocrine signalling (Figure 2i), whose combined action is necessary for developmental progression (Koyama et al., 2020; Liu et al., 2018; Yamanaka et al., 2013). However, *Sirt6* dependent causal/consequential interactions between these pathways need to be investigated in future.

### 2.3 Adult *Drosophila Sirt6* mutants display ageing‐associated physiological deficits

**FIGURE 3:**
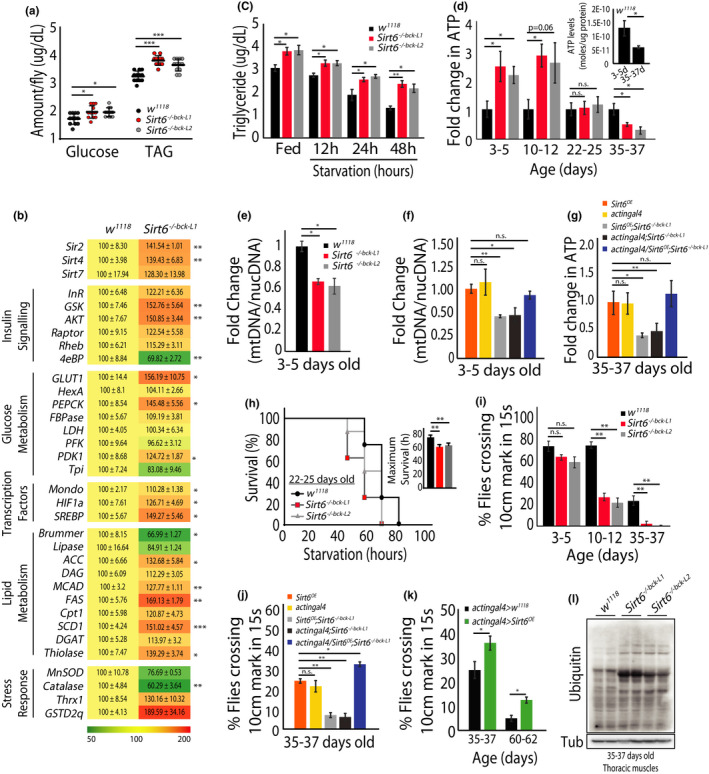
Adult *Drosophila Sirt6* mutants display ageing‐associated physiological deficits. (a) Total glucose and TAG levels in 3–5 day old *w^1118^* and *Sirt6^−/−bck−L1/L2^* flies (N = 3, n = 10 with 8 flies per n). (b) Real‐time PCR analysis of gene expression in 3–5 day old *w^1118^* and *Sirt6^−/−bck−L1/L2^* flies (N = 2, n = 3 with 8 flies per n). (c) Relative whole body TAG levels at fed and starved (12, 24 and 48 h) in 3–5 day old *w^1118^* (control) and *Sirt6^−/−bck−L1/L2^* flies (N = 2, n = 4 with 8 flies per n). (d) Whole body ATP levels across ages in *w^1118^* and *Sirt6^−/−bck−L1/L2^* flies. Inset depicts age‐associated change in total ATP levels in *w^1118^* flies (N = 3, n = 6 with 10 flies per n). (e) Mitochondrial DNA content (normalised to nuclear DNA) in 3–5 days old *w^1118^* and *Sirt6^−/−bck−L1/L2^* flies (N = 2, n = 3 with 30 flies per n). (f) Mitochondrial DNA content (normalised to nuclear DNA) in 3–5 days old flies, as per indicated genotypes. (N = 2, n = 3 with 30 flies per n). (g) Whole body ATP levels in 35–37 days old flies, as per indicated genotypes (N = 2, n = 3 with 30 flies per n). (h) Starvation survival in 22–25 day old *w^1118^* and *Sirt6^−/−bck−L1/L2^* flies (N = 3, n = 6 with 10 flies per n). (i) Percentage of *w^1118^* and *Sirt6^−/−bck−L1/L2^* flies crossing the 10 cm mark in 15 s, across ages, as indicated. (N = 4, n = 80–100). (j) Rescue of physical activity by transgenic expression of *Sirt6* in old *Sirt6^−/−bck−L1/L2^* flies (N = 4, n = 80–100). (k) Assessment of physical activity in 35–37 and 60–62 day old flies overexpressing *Sirt6* on a wild‐type background (N = 4, n = 80–100). (l) Representative immunoblot of total ubiquitinated proteins in 35–37 day old thoracic muscles of *w^1118^* and *Sirt6^−/−bck−L1^* ^/^ *^L2^*. All data presented are mean ±s.e.m. Asterisk depicts *p* values (*p < 0.05, **p < 0.01 and ***p < 0.001) as observed by Student's *t*‐test or two‐way ANOVA, as applicable

Mammalian SIRT6 has been demonstrated as an important factor regulating physiological homeostasis, and its absence has been shown to accelerate ageing (Mostoslavsky et al., 2006; Tasselli et al., 2017). Hence, we next set out to investigate metabolic fitness of adult *Sirt6* ^−/−^ *^bck^* ^−^ *^L1^* ^/^ *^2^* flies, across ages. At baseline, total glucose and triglyceride (TAG) levels were higher in 3–5 day old *Sirt6* ^−/−^ *^bck^* ^−^ *^L1^* ^/^ *^2^* flies compared to *w^1118^* controls (Figure 3a). This was associated with changes in the expression of genes involved in glucose and lipid metabolism, and stress response (Figure 3b). Notably, this was consistent with the ability of mammalian SIRT6 to orchestrate metabolic gene program (Chang et al., 2020; Tasselli et al., 2017). However, absence of *Sirt6* did not seem to affect organismal survival in response to starvation and oxidative stress (Figure S3a,b). Intriguingly, the lack of resistance to starvation survival was observed despite high levels of TAGs at baseline and during starvation in *Sirt6* ^−/−^ *^bck^* ^−^ *^L1^* ^/^ *^2^* flies (Figure 3c).

This prompted us to assess the energetic status of these flies and we found that while ATP was higher in young *Sirt6* ^−/−^ *^bck^* ^−^ *^L1^* ^/^ *^2^* (3–5 days old), there was a drastic decrease in 35–37 day old flies when compared to age‐matched controls (Figure 3d). To check if this was associated with a change in mitochondrial content, we measured levels of mitochondrial DNA (mtDNA) and expression of genes involved in mitochondrial biogenesis. Contrary to our expectations, there was a significant decrease in mtDNA (Figure 3e) and expression of *Spargel* and *Delg*, which are involved in mitochondrial biogenesis (Figure S3c). This was also associated with lower amounts of *TFAM*, *Cox4* and *ATP5α*, indicative of reduced mitochondrial content (Figure S3c,d). Whether this is due to higher energy production per mitochondria, decoupling of mitochondrial biogenesis and function or compensatory increase in glycolytic ATP production needs to be investigated in future. Nonetheless, unlike in young flies, aged *Sirt6* mutants displayed corroborated reduction in both ATP (Figure 3d) and mitochondrial content (Figure S3e). Notably, both mtDNA and ATP levels in old *Sirt6* ^−/−^ *^bck^* ^−^ *^L1^* ^/^ *^2^* flies were rescued by transgenic *Sirt6* expression (Figure 3f,g; Figure S3f). Interestingly, unlike in young flies, assaying for starvation survival of 22–25 day old adults showed poor resistance in *Sirt6* ^−/−^ *^bck^* ^−^ *^L1^* ^/^ *^2^* flies (Figure 3h). This raises the possibility of age‐dependent interplay between energetics and starvation survival, that is governed by *Sirt6*.

Our results hinted at perturbed physiological homeostasis in the *Sirt6* ^−/−^ *^bck^* ^−^ *^L1^* ^/^ *^2^* flies, which combined with reports in mammals (Chang et al., 2020; Mostoslavsky et al., 2006; Tasselli et al., 2017), motivated us to ask if this had any impact on ageing. We found an age‐associated decline in negative geotactic activity that became evident at 10–12 days of age and worsened in 35–37 day old flies (Figure 3i). Interestingly, not only did transgenic expression of *Sirt6* rescue this phenotype in old flies (Figure 3J), overexpression of *Sirt6* on a control background also proved to be beneficial in improving age‐associated loss in physical activity (Figure 3k).

Earlier reports have used global poly‐ubiquitination of muscle proteins as a measure of proteostatic stress and decline in muscle function (Demontis & Perrimon, 2010). As anticipated and shown earlier in mammals (Roichman et al., 2021), we observed a dramatic increase in protein ubiquitination in muscle lysates of *Sirt6* ^−/−^ *^bck^* ^−^ *^L1^* ^/^ *^2^* flies at 35–37 days of age (Figure 3l).

Further, fecundity is used as one of the parameters to investigate physiological fitness (Barnes et al., 2008). To score if presence or absence of *Sirt6* affected fecundity, we estimated the number of eggs laid in heterologous crosses, as indicated (Figure S3g). We found a significant reduction in the number of eggs laid, independent of whether *Sirt6* was genetically perturbed in the male or the female fly (Figure S3g). While exciting, this reduced fecundity could possibly be attributed to multiple factors, from metabolic to epigenetic mechanisms, which prompts further investigation.

Together, these results not only posit a central role for *Sirt6* in regulating invertebrate physiology but also highlight its evolutionarily conservation in dictating, age‐dependent fitness and healthspan.

### 2.4 Absence of *Sirt6* reduces lifespan in *Drosophila*

**FIGURE 4:**
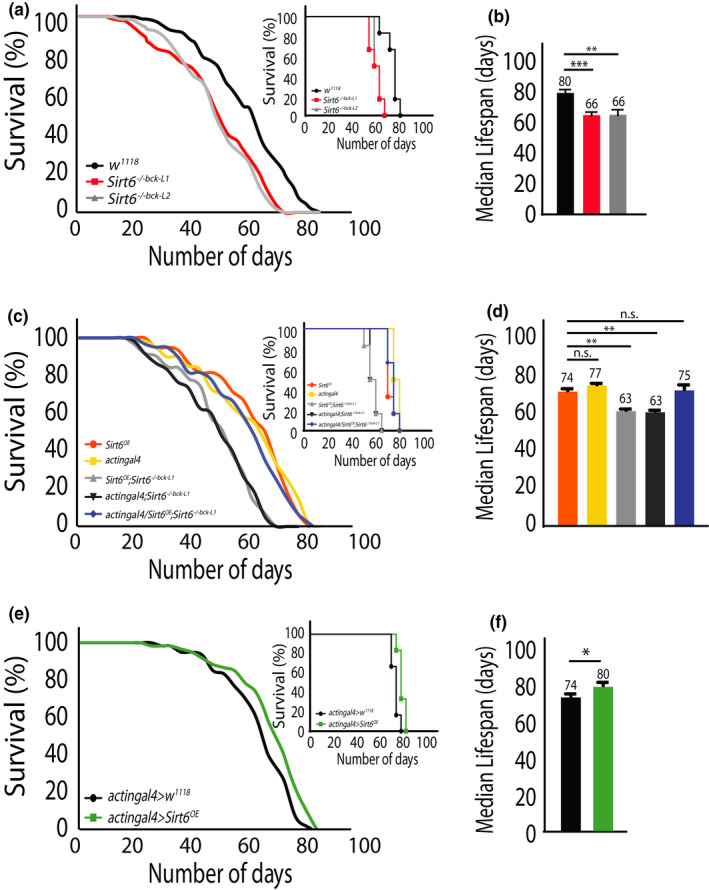
Absence of *Sirt6* reduces lifespan in *Drosophila*. (a) Representative life spans of *w^1118^* and *Sirt6^−/−bck−L1/L2^* flies on normal diet. Inset depicts log‐rank (Mantel‐Cox) survival curve for indicated genotypes (n = 10 with 10 flies per n). (b) Median life spans of *w^1118^* and *Sirt6^−/−bck−L1^* ^/^ *^L2^* flies (N = 3, n = 10 with 10 flies per n). (c) Representative life spans for transgenic rescue of *Sirt6* in mutant flies. Inset depicts log‐rank (Mantel‐Cox) survival curve for indicated genotypes (n = 10 with 10 flies per n). (d) Median lifespans of controls and transgenic *Sirt6* rescue flies (N = 3, n = 10 with flies per n). (e) Life spans of control and transgenic *Sirt6* overexpressing flies (n = 10 with 10 flies per n). Inset depicts log‐rank (Mantel‐Cox) survival curve for indicated genotypes. (f) The median life spans of control and transgenic *Sirt6* overexpressing flies (N = 3, n = 10 with flies per n). Log‐rank (Mantel‐Cox) test was used to plot survival curves and statistical analysis. Student's *t*‐test and two‐way ANOVA were used to analyse statistical significance of the data (*p < 0.05, **p < 0.01 and ***p < 0.001)

The physiological defects observed in *Sirt6* ^−/−^ *^bck^* flies and previous studies on mammals (Chang et al., 2020; Mostoslavsky et al., 2006; Tasselli et al., 2017), prompted us to investigate the role of *Sirt6* in invertebrate ageing. Survival analysis of backcrossed *Sirt6* ^−/−^ *^bck^* flies (both Line 1 and 2) displayed a significant reduction in lifespan of the mutant flies (Figure 4a,b). Importantly, when compared to *w^1118^* controls, *Sirt6* mutant flies displayed a reduction in both maximum as well as median lifespan (Figure 4a,b). To further validate the potential role of *Sirt6* in mediating lifespan, we rescued its expression using *actingal4*/*Sirt6^OE^*; *Sirt6* ^−/−^ *^bck^ flies*. As shown in Figure 4c,d, genetic rescue of *Sirt6* restored both the maximum and median lifespan in mutant flies, which was comparable to controls. By employing two independent mutant lines, which were backcrossed to the *w^1118^* control line and demonstrating a genetic rescue that restores lifespan deficits, we rule out potential contribution by background genetic mutations. Interestingly, overexpression of *Sirt6* (*actingal4*/*Sirt6^OE^*) resulted in a small but significant increase in lifespan of control flies (Figure 4e,f). These results clearly demonstrated that similar to its mammalian counterpart (Mostoslavsky et al., 2006; Roichman et al., 2021; Tasselli et al., 2017), *Sirt6* in flies is required for organismal survival and highlighted its evolutionarily conserved role in regulating lifespan in both invertebrates and vertebrates.

### 2.5 *Sirt6* couples differential nutrient availability to larval development

In addition to regulating larval growth, nutrient inputs during early development, also determine adult physiological fitness (Grangeteau et al., 2018; Rehman & Varghese, 2021; Tu & Tatar, 2003). However, mechanisms that link developmental/metabolic cues to adult physiology remain elusive. Given the dependence of larval growth on *Sirt6*, we were curious to investigate if/how loss of *Sirt6* affected the emergence of altered metabolic phenotypes in adulthood. Specifically, we wanted to ascertain the interplay between larval nutrient availability and *Sirt6*.

**FIGURE 5:**
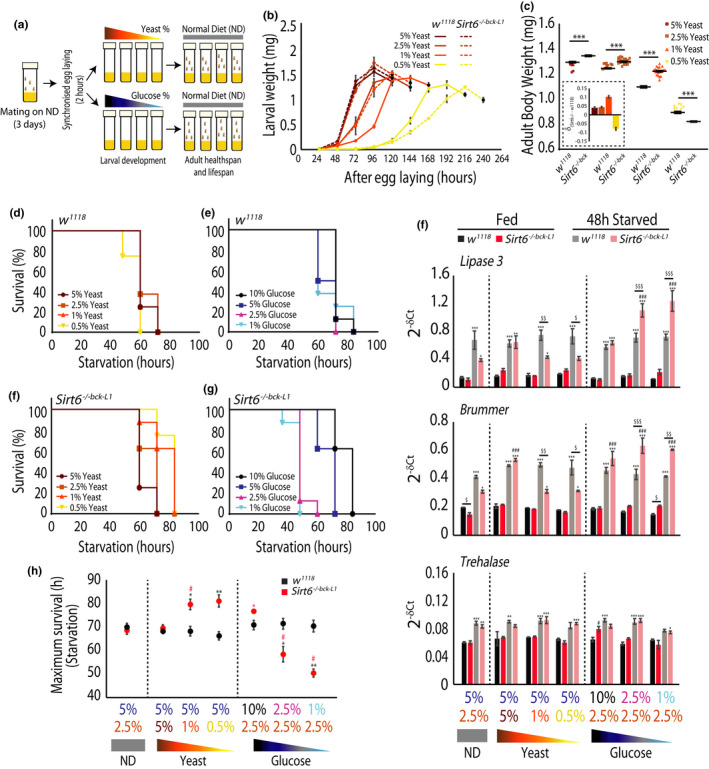
*Sirt6* is essential for coupling developmental nutrient availability to adult fitness. (a) Schematic of experimental paradigm used for larval diet perturbation with varying concentrations of yeast and glucose, as indicated. (b) Weight gain and pupation onset (marked in black) in *w^1118^* (control) and *Sirt6^−/−bck−L1^* larvae reared on differential yeast concentrations, as indicated. (N = 3, n = 20–25). (C) Body weight measurements in 3–5 day old *w^1118^* and *Sirt6^−/−bck−L1^* flies (N = 3, n = 20–25 per genotype). Inset depicts change in body weight between *w^1118^* and *Sirt6^−/−bck−L1^* flies across yeast concentrations. (d, e) Representative plot for starvation survival in 3–5 day old *w^1118^* flies reared under differential concentration of yeast (d) and glucose (e) diets, as indicated (n = 8 with 10 flies per n). (f, g) Representative plot for starvation survival in 3–5 day old *Sirt6^−/−bck−L1^* flies reared under differential concentrations of yeast (f) and glucose (g) diets, as indicated (n = 8 with 10 flies per n). (h) Maximum survival under starvation in *w^1118^* and *Sirt6^−/−bck−L1^* flies reared on differential concentrations of yeast and glucose diets, from three independent experiments. Asterisks indicate comparison with *w^1118^* grown on ND and hashtags depict comparison between *w^1118^* and *Sirt6^−/−bck−1^* for the particular diet, as indicated. (N = 3, n = 8 with 10 flies per n). (i) Quantitative PCR analysis for change in gene expression post 48 h of starvation in 3–5 day old *w^1118^* and *Sirt6^−/−bck−L1^* flies, gown under differential concentration of yeast and glucose, as indicated. ^$^statistical significance between *w^1118^* and *Sirt6^−/−bck−L1^* flies under both fed (black vs. red) and in response to 48 h starvation (grey vs. pink), within a diet group. ^*^statistical significance between fed and 48 h starved flies for each genotype (black vs. grey and red vs. pink), within a diet group. ^#^statistical significance with respect to control diet (ND) across diet regimes (comparison within each coloured cohort). Student's *t*‐test and two‐way ANOVA were used to analyse statistical significance of the data (*, ^#^, ^$^ *p* < 0.05, **, ^##^, ^$$^ *p* < 0.01 and ***, ^###^, ^$$$^ *p* < 0.001)

To this extent, following timed egg laying, control *w^1118^* and *Sirt6* ^−/−^ *^bck^* ^−^ *^L1^* were reared on media with varying concentrations of glucose and yeast and, following eclosion, flies were grown on normal diet (ND; Figure 5a). This paradigm allowed us to assess not only nutrient‐dependent impact on larval development but also investigate consequent physiological plasticity in adulthood. As reported earlier (Güler et al., 2015; Reis, 2016), we found developmental delay upon yeast limitation (keeping all other components same as in ND), in *w^1118^* flies (Figure S4a). Interestingly, on assaying for the effect of glucose titration on development, *w^1118^* control larvae showed a bidirectional change in weight gain and TTP (Figure S4b). We found accelerated and retarded developmental kinetics when *w^1118^* larvae were grown on low and high glucose concentrations, respectively. While these results are consistent with previous studies, which have employed different yeast to glucose ratios (Güler et al., 2015; Reis, 2016), our results unequivocally demonstrate the effect of glucose in the background of constant yeast/protein on larval development.

Absence of *Sirt6* led to yeast concentration‐dependent developmental delay viz. from 2.5% yeast (ND) to 1% and subsequently to 0.5% yeast (Figure 5b). Interestingly, at 0.5% yeast, *Sirt6* ^−/−^ *^bck^* ^−^ *^L1^* flies seemed to have lost the growth advantage with respect to *w^1118^* controls and developed at a much slower rate (Figure 5b). It was intriguing to find that at yeast concentration of 5% there was no difference in developmental kinetics in the *Sirt6* ^−/−^ *^bck^* ^−^ *^L1^* flies, neither when compared to ND (2.5% yeast) nor with respect to the corresponding *w^1118^* controls (Figure 5b).

Further, when the amount of glucose was altered, we did not find any phenotypic variation in *Sirt6* ^−/−^ *^bck^* ^−^ *^L1^* larvae, except when reared on media containing 10% glucose, which caused a delay (Figure S4c). This was not only distinct from *w^1118^* controls, which showed a bidirectional effect, but also indicated a loss of glucose‐dependent control of development in the absence of *Sirt6*.

### 2.6 *Sirt6* is essential for coupling developmental nutrient availability to adult fitness

Continuing on our efforts to delineate the interplay between *Sirt6* and developmental nutrient inputs, we next investigated its long‐term impact on adult fitness. Despite the dependence of larval growth on glucose availability during development, this did not seem to affect adult body weight in either the *w^1118^* controls or *Sirt6* ^−/−^ *^bck^* ^−^ *^L1^* flies (Figure S4d). On the other hand, larval yeast restriction led to a progressive decline in adult body weight in both *w^1118^* control and *Sirt6* ^−/−^ *^bck^* ^−^ *^L1^* alike. However, it was interesting to observe that *Sirt6* ^−/−^ *^bck^* ^−^ *^L1^* flies had a significantly higher body mass except when larvae were grown on 0.5% yeast (Figure 5c).

Next, we asked if these differential growth rates and adult body weights also impinged on healthspan parameters and resistance to adult stresses. Our results demonstrate that independent of the nutrient inputs during larval development, adult control *w^1118^* flies were equally resistant to starvation stress (Figure 5d,e; Figure S4e). Specifically, neither larval glucose nor yeast restriction had any effect on starvation survival in wild‐type adults (Figure 5d,e; Figure S4e). Surprisingly, in the absence of *Sirt6*, larval yeast restriction seemed to provide survival advantage in response to starvation stress in adults (Figure 5f; Figure S4e). However, larval glucose restriction in *Sirt6* ^−/−^ *^bck^* ^−^ *^L1^* significantly decreased tolerance to starvation (Figure 5g; Figure S4f). This starvation sensitivity was not only apparent when compared to *w^1118^* flies, whose larvae were grown on media containing same glucose concentration (Figure 5h), but also within the mutants, which were exposed to elevated levels of glucose during development (Figure 5g,h; Figure S4e).

Earlier reports have shown deregulated expression of genes involved in carbohydrate (Trehalose) and lipid metabolism as a contributing factor governing starvation survival. In this regard, we found that while genes involved in lipid metabolism were significantly altered, expression of *Trehalase* remained unaffected between control *w^1118^* and *Sirt6* ^−/−^ *^bck^* ^−^ *^L1^* flies. Specifically, unlike *w^1118^*, levels of both *Lipase3* and *Brummer* were opposingly regulated in *Sirt6* ^−/−^ *^bck^* ^−^ *^L1^*, based on the developmental nutrition inputs. To elaborate, *Sirt6* ^−/−^ *^bck^* ^−^ *^L1^* flies reared on limiting concentrations yeast, which showed increased starvation survival (Figure 5f) had poor induction of *Lipase3* and *Brummer*. Conversely, mutant flies grown on media with limited glucose displayed enhanced induction and poor starvation survival (Figure 5g,i).

**FIGURE 6:**
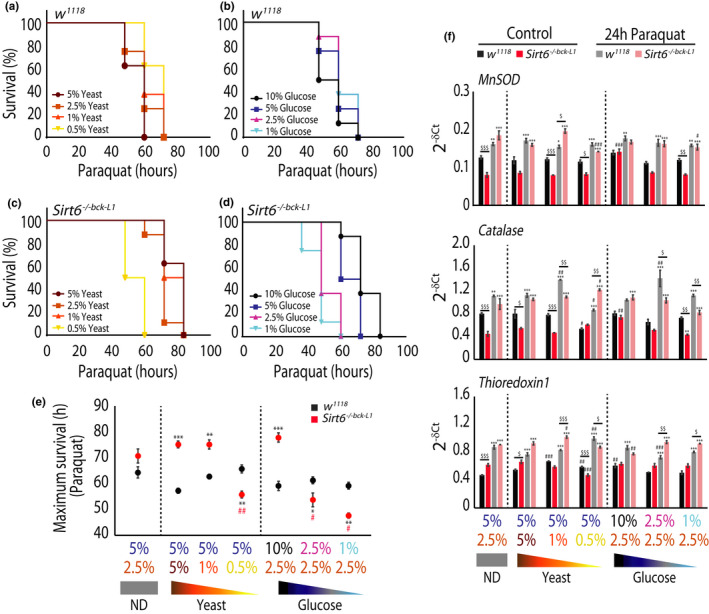
*Sirt6* is essential for coupling developmental nutrient availability to adult fitness. (a, b) Representative plot for oxidative stress survival on 20 mM Paraquat in 3–5 day old *w^1118^* flies reared under differential concentrations of yeast (a) and glucose (b) diets, as indicated (n = 8 with 10 flies per n). (c, d) Oxidative stress survival on 20 mM Paraquat in 3–5 day old *Sirt6^−/−bck−L1^* flies reared under differential concentrations of yeast (c) and glucose (d) diets, as indicated (n = 8 with 10 flies per n). (e) Maximum survival under oxidative stress in *w^1118^* and *Sirt6^−/−bck−L1^* flies reared on differential concentrations of yeast and glucose diets, from three independent experiments. Asterisks indicate comparison with *w^1118^* grown on ND and hashtags depict comparison between *w^1118^* and *Sirt6^−/−bck−1^* for the particular diet, as indicated. (N = 3, n = 8 with 10 flies per n). (f) Quantitative PCR analysis for change in gene expression post 24 h of 20 mM paraquat exposure in 3–5 day old *w^1118^* and *Sirt6^−/−bck−L1^* flies, gown under differential concentration of yeast and glucose, as indicated. ^$^statistical significance between *w^1118^* and *Sirt6^−/−bck−L1^* flies for both control (black vs. red) and in response to 24 h paraquat treatment (grey vs. pink), within a diet group. *Statistical significance between control and 24 h paraquat treated flies for each genotype (black vs. grey and red vs. pink), within a diet group. ^#^Statistical significance with respect to control diet (ND) across diet regimes (comparison within each coloured cohort). Student's *t*‐test and two‐way ANOVA were used to analyse statistical significance of the data (*, ^#^, ^$^ *p* < 0.05, **, ^##^, ^$$^ *p* < 0.01 and ***, ^###^, ^$$$^ *p* < 0.001)

Next we wanted to ask if *Sirt6* was required for coupling developmental nutrition to adult oxidative stress resistance, especially since we found (a) reduced expression of ROS scavenging enzymes, *MnSOD* and *Catalase* in *Sirt6* ^−/−^ *^bck^* ^−^ *^L1^* flies and (b) no difference in oxidative stress survival, when grown on ND (Figure 3b; Figure S3a). We found, while larval yeast restriction conferred some advantage towards oxidative stress (Figure 6a; Figure S4f), glucose titrations had no impact on oxidative stress tolerance in *w^1118^* control flies (Figure 6b; Figure S4f).

On the other hand, rearing *Sirt6* ^−/−^ *^bck^* ^−^ *^L1^* larvae on either high yeast or high glucose diets, lead to better oxidative stress resistance in adulthood with significant enhancement in survival following paraquat treatment, when compared to controls (Figure 6c–e; Figure S4f). However, we found both larval yeast as well as glucose restriction significantly increased the susceptibility to oxidative stress in*Sirt6* ^−/−^ *^bck^* ^−^ *^L1^* flies (Figure 6c–e; Figure S4f). Gene expression analysis also revealed differential induction of genes in response to oxidative stress between control *w^1118^* and *Sirt6* ^−/−^ *^bck^* ^−^ *^L1^* adult flies, in a larval nutrition dependent manner (Figure 6f).

Together, these results clearly posit that plasticity and potential reprogramming following differential nutrient inputs during development are governed by *Sirt6* with implication on adult stress survival.

### 2.7 *Sirt6* is essential for coupling developmental nutrient availability to lifespan

Our results on healthspan and stress resistance also motivated us to investigate if larval nutrition impinged on adult lifespan. As reported earlier (May et al., 2015), larval yeast restriction caused a progressive increase in both median and maximal lifespans in control *w^1118^* flies (Figure S5a), which was blunted in *Sirt6* ^−/−^ *^bck^* ^−^ *^L1^*. Notably, absence of *Sirt6* affected both median (81 days vs. 73 days when larvae were reared on 1% yeast and 86 days vs. 77 days for 0.5% yeast) and maximum lifespans (84 days vs. 76 days when larvae were reared on 1% yeast and 92 days vs. 84 days for 0.5% yeast) when compared to controls (Figure S5b,e). Unlike the impact of yeast restriction during development, glucose variations in larval diet did not seem to alter adult lifespan (Figure S5c–e) for either of the genotypes. Taken together, our results demonstrated the importance of *Sirt6* in coupling larval development to adult fitness and lifespan, in a nutrient‐dependent manner.

## 3 DISCUSSION

Organismal development, health and survival are dictated by nutrient composition and availability. Owing to the obvious relevance for human health, studies over decades have tried to unravel physiological changes and underlying mechanisms that dictate diet‐dependent effects on organismal health, which are evolutionarily conserved (Koyama et al., 2020; May et al., 2015). For example, such efforts have revealed the benefits of dietary/calorie restriction, especially in adults (Good & Tatar, 2001; Lee et al., 2006; Zheng et al., 2005). Further, developmental nutrition has been shown to be one of the primary governing factors behind early resource uptake, allocation and utilisation (May et al., 2015; Palgunow et al., 2012; Rehman & Varghese, 2021). Despite these, genetic and molecular factors that integrate developmental nutritional cues and determine physiological fitness in adults, are limited to studies in insulin signalling and its interplay with downstream components like FOXO/*daf*‐*2* (Koyama & Mirth, 2016; Murphy & Hu, 2018). In this context, our current study not only highlights the evolutionarily conserved role of *SIRT6* in regulating ageing, but more importantly identifies it as a key factor that couples developmental nutrition to larval growth and consequently, adult physiology.

Sirtuins in *Drosophila* have been identified as important metabolic sensors essential for maintaining physiological homeostasis, stress resistance and survival (Banerjee et al., 2012, 2013, 2017; Frankel et al., 2011; Parik et al., 2018; Sejour et al., 2020; Wood et al., 2018). Our current findings employing loss of function CRISPR mutants for *Sirt6* and genetic rescue clearly demonstrate its evolutionarily conserved role in determining adult lifespan. This is consistent with a study by Kusama et al., which identified *CG6284* as a sirtuin homologue whose suppression resulted in reduced lifespan (Kusama et al., 2006). Besides impinging on lifespan, we illustrate that *Sirt6* maintains glucose and TAG homeostasis, and importantly its absence leads to exacerbated age‐associated decline in physical activity. Genetic rescue of *Sirt6* or a gain of function expression in the whole body not only provide conclusive evidence but raises the possibility of targeting mechanisms that activate or inhibit *Sirt6* as potential intervention to mitigate age‐associated decline in physiology.

Our serendipitous findings further demonstrate the crucial role of *Sirt6* in governing larval development and pupariation kinetics. Attainment of critical weight is a crucial developmental checkpoint in insects, which couples larval growth rate and maturation to nutritional availability. Previous reports have invoked fat body‐prothoracic gland axis and TOR/insulin signalling in regulating developmental pathways such as ecdysone signalling (Koyama et al., 2020). Our preliminary data hint towards potential involvement of pathways that dictate larval size, critical weight, and insulin and ecdysone signalling. Given the importance of these signalling pathways in adult fitness and lifespan (Simon et al., 2003), it will be particularly interesting to address the *Sirt6*‐*ecdysone* interplay in this context. Nonetheless, these are significant findings for multiple reasons, including since loss of sirtuins across model systems, have not been associated with accelerated or over‐growth during development. More importantly, genetic perturbations of master regulators of nutrient sensing such as AMPK and TOR have been associated with developmental lethality or gross deficits (Bland et al., 2010; Radimerski et al., 2002). Therefore, owing to the crucial requirement of dietary inputs in regulating development, our study not only posits *Sirt6* as a key component, but will also likely motivate further research in elucidating genetic/molecular pathways that link metabolism with early growth, at an organismal level.

Significantly altered carbohydrate to protein inputs in case of humans as well as unpredictable nutrition availability and ill‐defined compositions in the wild have been associated with developmental perturbations (de Brito Alves & Costa‐Silva, 2018; Delpierre et al., 2016; Hibshman et al., 2016; Jahan‐Mihan et al., 2015; Parlee & MacDougald, 2014; Watkins & Sinclair, 2014). Independent efforts have shown that metabolic and reproductive fitness are intrinsically dependent upon dietary macronutrient ratios and environmental variations (Behrman et al., 2015; Brankatschk et al., 2018; Klepsatel et al., 2020). In addition to these reports, detrimental effects of both over‐ and under‐nutrition in early stages of life are well documented (Martins et al., 2011; May et al., 2015). Despite these, evolutionarily conserved mechanisms that may either buffer or exacerbate the impact of skewed carbohydrate to protein ratios in the diet, on development, remain elusive. In this regard, we have uncovered differential impact of yeast/protein and glucose in regulating larval growth and adult physiology, and their dependence on *Sirt6*. Strikingly, unlike yeast titrations, altering glucose concentrations led to a bidirectional change in development with respect to TTP in wild‐type larvae. We found that presence of *Sirt6* is crucial to integrate diet‐dependent changes with development as *Sirt6* ^−/−^ *^bck^* ^−^ *^L1^* larvae failed to modulate their growth with changing nutrition conditions. For example, at low concentrations of yeast (0.5%), *Sirt6* mutants lost their developmental advantage over *w^1118^* controls.

We further demonstrate the essential role of *Sirt6* in mitigating adult stress, which emerges as a consequence of differential nutrient inputs during larval growth. Specifically, we employed starvation and paraquat treatments to score for response to metabolic and oxidative stresses, which are used as healthspan measures. Upon starvation, loss of *Sirt6* clearly led to reduced survival in flies whose larvae were reared under low yeast/protein and glucose‐containing diets. Surprisingly, a similar loss of fitness was observed when *Sirt6* ^−/−^ flies were subjected to oxidative stress, following paraquat treatment. Even though excess calorie inputs in adults have been earlier shown to reduce resistance to oxidative stress (Zheng et al., 2005), our results illustrate a long‐lasting effect of larval nutrition in determining organismal response to paraquat treatment. Together, these indicate that *Sirt6* is necessary to couple larval nutrition to adult healthspan. Whether this is contributed by the ability of *Sirt6* to epigenetically reprogram gene expression to shield adult physiological fitness from variations in larval nutrient inputs, needs to be addressed in future.

Earlier studies on mammalian SIRT6 had demonstrated that it regulates a plethora of genes and thus impacts organismal physiology (Chang et al., 2020; Mostoslavsky et al., 2006; Tasselli et al., 2017). The phenotypes described in our study, especially metabolic and energy homeostasis and age‐associated decline in fitness could be a consequence of distinct transcriptional cascades being controlled by *Sirt6* in flies. However, based on all our results, we speculate that its role in regulating mitochondrial functions might be central. On the other hand, the underlying mechanisms that couple developmental nutrition to adult fitness in a *Sirt6* dependent manner, are still unclear. In addition, given that *Sirt6* seems to govern both developmental as well as adult phenotypes, it will be crucial to decipher its role at different time windows during the lifetime of an organisms as well as tissue‐specific contributions.

In conclusion, our results highlight emergence of nutrient‐dependent life‐history traits and identify *Sirt6* as a key molecular/genetic factor. These findings not only invoke a regulatory role for *Sirt6* during development, which was hitherto unknown, it also raises a possibility of its function in governing macronutrient‐dependent (yeast vs. glucose) plasticity during larval growth and subsequent stress resistance in adulthood. Given that our understanding of physiological plasticity and memory, which is encoded by dietary inputs throughout life is still poor, the current study posits the existence of nutrient‐dependent thresholds that exert a control over physiological fitness.

## 4 EXPERIMENTAL PROCEDURES

### 4.1 Fly strains

*w^1118^*, *dSir2* null (BL8838) and *Sirt4* null (BL8840) flies were obtained from Bloomington Stock Centre (Indiana University, USA). All flies used in the study were backcrossed to *w^1118^* for eight generations. a*ctingal4* (w*; P{Act5C‐GAL4}25FO1/CyO) was a kind gift from Narasimha lab, TIFR Mumbai. All adult fly experiments were performed on females.

#### 4.1.1 Generation of CRISPR *Sirt6* mutant (*Sirt6^−/−^^bck^*)

CRISPR‐mediated mutagenesis was done by WellGenetics Inc. using modified methods of (Kondo and Ueda 2013). In brief, gRNA sequences ATGAGCTGCAACTACGCGGA[TGG] and ATCCGCGTAGT TGCAGCTCA[TGG] were cloned into a U6 promoter plasmid. Cassette Stop‐RFP containing 3‐frame stop codons and 3xP3‐RFP and 1046bp upstream homology arm and 745bp downstream homology arm were cloned into pUC57‐Kan as donor template for repair. *CG6284*‐targeting gRNAs and hs‐Cas9 were supplied in DNA plasmids, together with donor plasmid for microinjection into embryos of control strain *w^1118^*. F1 flies carrying selection marker of 3xP3‐RFP were further validated by genomic PCR and sequencing and two independent lines were developed. CRISPR generated a 62‐bp deletion allele of *CG6284*, deleting around ATG region of CG6284 gene and is replaced by cassette Stop‐RFP. PCR verification was performed using primers (highlighted in yellow) as mentioned in Figure S1c.

#### 4.1.2 Generation of transgenic *UAS‐Sirt6* (*Sirt6^OE^*) fly

d*Sirt6* was amplified from cDNA of *w^1118^* flies using forward primer 5′ TCTGCGGCCGCGGTACCATGAGCTGCAACTACGCGGATGGATTG 3′ and reverse primer 5′ GCGTCTAGACTCGAGTTACTTGTCATCGTCGTCCTTGTAGTCCGTGTACTTTGTTTTCTTAGCTTTAG 3′ containing a FLAG tag and cloned into pUAST‐attB plasmid. This plasmid was used for generating transgenic flies at the Fly Facility at C‐CAMP, India.

#### 4.1.3 Fly lines used for *Sirt6* rescue and overexpression

*UAS*‐*Sirt6* and *actingal4* lines were crossed to Sirt6^−/−bck−L1^ to generate homozygous *Sirt6^OE^*; *Sirt6* ^−/−^ *^bck^* ^−^ *^L1^* and *actingal4*; *Sirt6* ^−/−^ *^bck^* ^−^ *^L1^*. These two homozygous lines were used as “Sirt6 mutant” controls and were crossed together to genetically rescue Sirt6 (*actingal4*/*Sirt6^OE^*; *Sirt6* ^−/−^ *^bck^* ^−^ *^L1^*).

### 4.2 Growth conditions

Flies were maintained under non‐crowding conditions on standard cornmeal diet and grown at 25°C with 12 h light/dark cycle. Age‐matched non‐virgin flies were used for all experiments. For larval experiments, synchronised populations were obtained by 2‐h timed egg laying of young mated females and care was taken to ensure equal number of eggs/larvae are present per vial.

### 4.3 Fly diets

Composition of standard cornmeal media (ND)‐5 g dextrose, 2.5% yeast extract, 8.6% cornmeal, 2% agar and 0.1% ortho‐phosphoric and propionic acids, each. For dietary perturbations, yeast or glucose concentrations were changed (0.5%, 1%, 2.5% and 5% or 1%, 2.5%, 5% and 10%, respectively, as mentioned in figure) keeping all other components constant.

### 4.4 Larval staging

Mated young female flies were put for egg laying for 2 h and larvae were scored at indicated time points (24, 48 and 72 h). Larval images were captured on Zeiss Stereo Discovery with AxioCam using 1× objective and at 12.5× zoom.

### 4.5 Pupation

Mated young female flies were put for egg laying for 2 h, and larvae were allowed to grow up to the wandering stage under normal growth conditions. Number of pupa per vial were scored every 6 h.

### 4.6 Wing dimensions

Dissected wings were imaged under a dissection microscope and imaged at 2× magnification. Wing dimensions were noted as previously described (Lack et al., 2016) and computed using Fiji‐ImageJ software.

### 4.7 Critical weight estimation

Critical weight was computed as previously described by Mirth et al. (2014). Briefly, individual control and *Sirt6* mutant larvae were weighed and placed in a 1.5 ml microfuge tube with a 10 × 50 mm strip of moist filter paper. TTP was recorded by checking the larvae every 4 h. Relationship between larval weight at starvation and TTP changes upon attaining critical weight which can be identified using a bi‐segmental linear regression as described previously by Muggeo (2003). Time to reach critical weight was obtained by fitting the critical weight into the larval growth curve.

### 4.8 Body weight measurements

Body weight measurements were done as previously described by (Van Voorhies et al., 2004). 10 flies (or 10–20 larvae) were pooled in a single micro‐centrifuge tube and snap‐frozen in liquid nitrogen. Body weight was measured using a fine balance.

### 4.9 NAD estimation

Total NAD levels were estimated by colorimetric detection using NAD/NADH quantification kit (Sigma, Cat. No. MAK037). Briefly, 40–60 mg larvae were snap‐frozen and lysed using extraction buffer. Lysate was spun at 14,000 rpm for 5 minutes at 4°C. Supernatant was deproteinised by passing through 10 kDa cut‐off spin filter, and the 10 µl eluent was used for NAD estimation as per manufacturer's protocol.

### 4.10 RNA isolation and reverse transcription

Total RNA was extracted from flies/larvae (8 flies/8–20 larvae) using TRIzol method (Ambion cat. no. 15596026). Briefly, homogenised fly samples were chloroform extracted by centrifuging at 12,000 rpm for 15 min at 4°C. The aqueous phase containing RNA was treated with isopropanol and incubated at room temperature for 10 min for precipitation. Precipitated RNA was spun down by centrifuging at 12,000 *g* at 4°C for 5 min. RNA pellet was washed in 75% ethanol and resuspended in DEPC‐treated water. 1ug of RNA was used for cDNA synthesis using SuperScript RT kit (cat. no. 18091050; Invitrogen), as per manufacturers protocol.

### 4.11 Quantitative PCR

Real‐time PCR was done using manufacturer's instructions for KAPA SYBR mix (KAPA 4600) and run on Light Cycler 96 System. *rp49* was used as internal control for normalisation. Primer sequences used in this paper are available in Supporting Information.

### 4.12 Triglyceride and glucose extraction and estimation

Eight flies were snap‐frozen in liquid nitrogen and homogenised into 650 µl of 0.05% TBST using a pestle. The homogenate was incubated at 70°C for 15 min and spun at 12,000 rpm for 15 min at 4°C. Supernatant was collected and used for TAG/glucose estimation (at Shahbazker's Labs). Normalisation was done using total protein, as estimated from Bicinchoninic Acid (BCA) method.

### 4.13 Starvation assay

3–5 day old flies reared on standard cornmeal diet were transferred to 2% agar with a density of 10 flies/vial. Flies were transferred onto fresh agar every 12 h, and death was scored for till the entire population was dead.

### 4.14 Oxidative stress response

Three to five day old flies were starved for 3 h before transferring into vials (10 flies per vial) with filter paper soaked in 5% sucrose and 20 mM Paraquat. Flies were flipped onto fresh vials with sucrose and paraquat every 12 h, and number of dead flies were scored.

### 4.15 ATP measurements

Five to six flies grown under non‐crowding conditions were snap‐frozen in liquid nitrogen at appropriate ages. Frozen flies were crushed to homogeneity in boiling water using a pestle and incubated at 95°C for 15 min. The homogenate was spun at 12,000 rpm for 7 min at 4°C, and the supernatant was used for ATP estimation using kit‐based method (Sigma FLAAM. cat. no. 32160414). Standard curve with known ATP concentrations was used to extrapolate the luminescence values, and protein estimation using a BCA kit (Pierce Thermofischer cat. no. 23225) was used for normalisation.

### 4.16 Mitochondrial DNA estimation

Total genomic DNA was isolated as described previously. For mitochondrial DNA estimation, total genomic DNA was isolated as described by Huang et al. (2009). The relative mitochondrial content was quantified by real‐time PCR using the primers for *COX* subunit I and nuclear *Actin*, for normalisation.

### 4.17 Negative geotaxis assay

Twenty to 25 age‐matched flies grown under non‐crowding conditions were immobilised on ice and transferred into graduated 25 cm glass vials. After a recovery period of 30 min, the flies were banged to the bottom of the tube and the tube was kept vertical to record climbing. Four trials were performed for each genotype with a 5‐min recovery period between each trial. Snapshots were taken from the video at 15 s, and number of flies which have crossed the 10 cm mark were recorded. All assays were performed at the same time of the day to avoid time of the day dependent variations in mobility.

### 4.18 Western Blotting

Eight to 10 flies were pooled and snap‐frozen in liquid nitrogen. Protein lysates were prepared by homogenising the flies on ice in RIPA (radio‐immunoprecipitation assay buffer) lysis buffer (50 mM Tris pH 8.0, 150 mM NaCl, 0.1% SDS [sodium dodecyl sulphate], 0.5% sodium deoxycholate, 1% Triton X‐100, 0.1% SDS, 1 mM sucrose, 1 mM PMSF (phenylmethylsulphonyl fluoride), protease inhibitor cocktail and phosphatase inhibitor—Sigma‐Roche 4906845001). The homogenate was centrifuged at 12,000 rpm for 15 min at 4°C to pellet insoluble debris. Supernatant was used for BCA estimation to compute protein concentration and loading buffer boiled lysate was used to resolve on 10% SDS‐PAGE. The resolved proteins were blotted onto PVDF membranes and probed with appropriate antibodies (anti‐Ubiquitin: Abcam‐ab7780 and anti‐β‐tubulin: Sigma‐Aldrich‐T8328). Chemiluminescence was detected using HRP (horseradish peroxidase)‐based reaction (Pierce, Thermo Scientific, cat. no. 32106) in Amersham Imager 600 system. Fiji‐ImageJ software was used for blot intensity measurements post‐background correction.

### 4.19 Fecundity analysis

Control and *Sirt6* mutant virgin females were mated with males (of both the genotypes, as indicated in Figure 1h) for three days. Mated females were separated from males into a fresh media vial and flipped every 2 days for 20 days. Average number of eggs laid per female over the 20‐day period was computed.

### 4.20 Lifespan assay

Three to five day old flies reared on cornmeal diet (with yeast/glucose variations, as mentioned in the figure legend) were transferred into fresh vials (medium composition as mentioned in figures) at a density of 10 flies/vial. The flies were flipped into fresh media containing vials every third day, and death was scored until the entire population was dead.

### 4.21 Statistical tests and analysis

Student's *t*‐test and two‐way ANOVA were used for estimating statistical significance, wherever applicable (*p < 0.05, **p < 0.01, ***p < 0.001). Graph Pad 8.0 was used for Log‐rank analysis of survival.

## CONFLICT OF INTEREST

The authors declare that no competing interests exist.

## AUTHOR CONTRIBUTIONS

Namrata Shukla and Ullas Kolthur‐Seetharam conceived the project, designed the experiments and performed data analysis. Namrata Shukla performed the experiments. Namrata Shukla and Ullas Kolthur‐Seetharam wrote the manuscript.

## Supporting information

Supplementary Material

## Data Availability

Data sharing is not applicable—no new data generated.

## References

[acel13576-bib-0001] Banerjee, K. K. , Ayyub, C. , Sengupta, S. , & Kolthur‐Seetharam, U. (2012). dSir2 deficiency in the fatbody, but not muscles, affects systemic insulin signaling, fat mobilization and starvation survival in flies. Aging, 4(3), 206–223. 10.18632/aging.100435 22411915 PMC3348481

[acel13576-bib-0002] Banerjee, K. K. , Ayyub, C. , Sengupta, S. , & Kolthur‐Seetharam, U. (2013). Fat body dSir2 regulates muscle mitochondrial physiology and energy homeostasis non‐autonomously and mimics the autonomous functions of dSir2 in muscles. Molecular and Cellular Biology, 33(2), 252. 10.1128/MCB.00976-12 23129806 PMC3554107

[acel13576-bib-0003] Banerjee, K. K. , Deshpande, R. S. , Koppula, P. , Ayyub, C. , & Kolthur‐Seetharam, U. (2017). Central metabolic sensing remotely controls nutrient‐sensitive endocrine response in *Drosophila* via Sir2/Sirt1–upd2–IIS axis. Journal of Experimental Biology, 220(7), 1187–1191. 10.1242/jeb.150805 28104798

[acel13576-bib-0004] Barnes, A. I. , Wigby, S. , Boone, J. M. , Partridge, L. , & Chapman, T. (2008). Feeding, fecundity and lifespan in female Drosophila melanogaster. Proceedings of the Royal Society B: Biological Sciences, 275(1643), 1675–1683. 10.1098/rspb.2008.0139 PMC2452982 18430646

[acel13576-bib-0005] Behrman, E. L. , Watson, S. S. , O'brien, K. R. , Heschel, M. S. , & Schmidt, P. S. (2015). Seasonal variation in life history traits in two *Drosophila* species. Journal of Evolutionary Biology, 28(9), 1691–1704. 26174167 10.1111/jeb.12690 PMC5089932

[acel13576-bib-0006] Blagosklonny, M. V. (2010). Revisiting the antagonistic pleiotropy theory of aging: TOR‐driven program and quasi‐program. Cell Cycle, 9(16), 3171–3176. 10.4161/cc.9.16.13120 20724817

[acel13576-bib-0007] Bland, M. L. , Lee, R. J. , Magallanes, J. M. , Foskett, J. K. , & Birnbaum, M. J. (2010). AMPK supports growth in *Drosophila* by regulating muscle activity and nutrient uptake in the gut. Developmental Biology, 344(1), 293–303. 10.1016/j.ydbio.2010.05.010 20478298 PMC2909368

[acel13576-bib-0008] Brankatschk, M. , Gutmann, T. , Knittelfelder, O. , Palladini, A. , Prince, E. , Grzybek, M. , Brankatschk, B. , Shevchenko, A. , Coskun, Ü. , & Eaton, S. (2018). A temperature‐dependent switch in feeding preference improves *Drosophila* development and survival in the cold. Developmental Cell, 46(6), 781–793. 10.1016/j.devcel.2018.05.028 30253170

[acel13576-bib-0009] Buhler, K. , Clements, J. , Winant, M. , Bolckmans, L. , Vulsteke, V. , & Callaerts, P. (2018). Growth control through regulation of insulin signalling by nutrition‐activated steroid hormone in *Drosophila* . Development, 145(21), dev165654. 30266830 10.1242/dev.165654

[acel13576-bib-0010] Chang, A. R. , Ferrer, C. M. , & Mostoslavsky, R. (2020). *SIRT6*, a mammalian deacylase with multitasking abilities. Physiological Reviews, 100(1), 145–169. 10.1152/physrev.00030.2018 31437090 PMC7002868

[acel13576-bib-0011] Danielsen, E. T. , Moeller, M. E. , & Rewitz, K. F. (2013). Nutrient signaling and developmental timing of maturation. Current Topics in Developmental Biology, 105, 37–67. 23962838 10.1016/B978-0-12-396968-2.00002-6

[acel13576-bib-0012] de Brito Alves, J. L. , & Costa‐Silva, J. H. (2018). Maternal protein malnutrition induced‐hypertension: New evidence about the autonomic and respiratory dysfunctions and epigenetic mechanisms. Clinical and Experimental Pharmacology and Physiology, 45(5), 422–429. 10.1111/1440-1681.12892 29164748

[acel13576-bib-0013] De Moed, G. H. , Kruitwagen, C. L. J. J. , De Jong, G. , & Scharloo, W. (1999). Critical weight for the induction of pupariation in Drosophila melanogaster: Genetic and environmental variation. Journal of Evolutionary Biology, 12(5), 852–858. 10.1046/j.1420-9101.1999.00103.x

[acel13576-bib-0014] Delpierre, C. , Fantin, R. , Barboza‐Solis, C. , Lepage, B. , Darnaudéry, M. , & Kelly‐Irving, M. (2016). The early life nutritional environment and early life stress as potential pathways towards the metabolic syndrome in mid‐life? A life course analysis using the 1958 British Birth cohort. BMC Public Health, 16(1), 1–19. 10.1186/s12889-016-3484-0 27538482 PMC4989336

[acel13576-bib-0015] Demontis, F. , & Perrimon, N. (2010). FOXO/4E‐BP signaling in *Drosophila* muscles regulates organism‐wide proteostasis during aging. Cell, 143(5), 813–825. 10.1016/j.cell.2010.10.007 21111239 PMC3066043

[acel13576-bib-0016] Frankel, S. , Ziafazeli, T. , & Rogina, B. (2011). dSir2 and longevity in *Drosophila* . Experimental Gerontology, 46(5), 391–396. 10.1016/j.exger.2010.08.007 20728527 PMC2997167

[acel13576-bib-0017] Gerofotis, C. D. , Kouloussis, N. A. , Koukougiannidou, C. , Papadopoulos, N. T. , Damos, P. , Koveos, D. S. , & Carey, J. R. (2019). Age, sex, adult and larval diet shape starvation resistance in the Mediterranean fruit fly: An ecological and gerontological perspective. Scientific Reports, 9(1), 1–12. 10.1038/s41598-019-47010-0 31341198 PMC6656776

[acel13576-bib-0018] Good, T. P. , & Tatar, M. (2001). Age‐specific mortality and reproduction respond to adult dietary restriction in Drosophila melanogaster. Journal of Insect Physiology, 47(12), 1467–1473. 10.1016/S0022-1910(01)00138-X 12770153

[acel13576-bib-0019] Grangeteau, C. , Yahou, F. , Everaerts, C. , Dupont, S. , Farine, J. P. , Beney, L. , & Ferveur, J. F. (2018). Yeast quality in juvenile diet affects *Drosophila melanogaster* adult life traits. Scientific Reports, 8(1), 1–9. 10.1038/s41598-018-31561-9 30166573 PMC6117321

[acel13576-bib-0020] Guerra, D. , Pezzoli, M. C. , Giorgi, G. , Garoia, F. , & Cavicchi, S. (1997). Developmental constraints in the *Drosophila* wing. Heredity, 79(6), 564–571. 10.1038/hdy.1997.200 9418264

[acel13576-bib-0021] Güler, P. , Ayhan, N. , Koşukcu, C. A. N. , & Önder, B. Ş. (2015). The effects of larval diet restriction on developmental time, pre‐adult survival, and wing length in *Drosophila melanogaster* . Turkish Journal of Zoology, 39(3), 395–403. 10.3906/zoo-1305-42

[acel13576-bib-0022] Hibshman, J. D. , Hung, A. , & Baugh, L. R. (2016). Maternal diet and insulin‐like signaling control intergenerational plasticity of progeny size and starvation resistance. PLoS Genetics, 12(10), e1006396. 10.1371/journal.pgen.1006396 27783623 PMC5081166

[acel13576-bib-0023] Hironaka, K. I. , Fujimoto, K. , & Nishimura, T. (2019). Optimal scaling of critical size for metamorphosis in the genus *Drosophila* . Iscience, 20, 348–358. 10.1016/j.isci.2019.09.033 31610371 PMC6817650

[acel13576-bib-0024] Houtkooper, R. H. , Pirinen, E. , & Auwerx, J. (2012). Sirtuins as regulators of metabolism and healthspan. Nature Reviews Molecular Cell Biology, 13(4), 225–238. 10.1038/nrm3293 22395773 PMC4872805

[acel13576-bib-0025] Huang, A. M. , Rehm, E. J. , & Rubin, G. M. (2009). Quick preparation of genomic DNA from *Drosophila* . Cold Spring Harbor Protocols, 2009(4), pp.pdb‐prot5198. 10.1101/pdb.prot5198 20147141

[acel13576-bib-0026] Jahan‐Mihan, A. , Rodriguez, J. , Christie, C. , Sadeghi, M. , & Zerbe, T. (2015). The role of maternal dietary proteins in development of metabolic syndrome in offspring. Nutrients, 7(11), 9185–9217. 10.3390/nu7115460 26561832 PMC4663588

[acel13576-bib-0027] Klepsatel, P. , Knoblochová, D. , Girish, T. N. , Dircksen, H. , & Gáliková, M. (2020). The influence of developmental diet on reproduction and metabolism in *Drosophila* . BMC Evolutionary Biology, 20(1), 1–15. 10.1186/s12862-020-01663-y 32727355 PMC7392729

[acel13576-bib-0058] Kondo, S. , & Ueda, R. (2013). Highly improved gene targeting by germline‐specific Cas9 expression in Drosophila. Genetics, 195(3), 715–721. 24002648 10.1534/genetics.113.156737 PMC3813859

[acel13576-bib-0028] Koyama, T. , & Mirth, C. K. (2016). Growth‐blocking peptides as nutrition‐sensitive signals for insulin secretion and body size regulation. PLoS Biology, 14(2), e1002392. 10.1371/journal.pbio.1002392 26928023 PMC4771208

[acel13576-bib-0029] Koyama, T. , Texada, M. J. , Halberg, K. A. , & Rewitz, K. (2020). Metabolism and growth adaptation to environmental conditions in Drosophila. Cellular and Molecular Life Sciences, 77, 4523–4551. 10.1007/s00018-020-03547-2 32448994 PMC7599194

[acel13576-bib-0030] Kusama, S. , Ueda, R. , Suda, T. , Nishihara, S. , & Matsuura, E. T. (2006). Involvement of *Drosophila* Sir2‐like genes in the regulation of life span. Genes & Genetic Systems, 81(5), 341–348. 10.1266/ggs.81.341 17159295

[acel13576-bib-0059] Lack, J. B. , Yassin, A. , Sprengelmeyer, Q. D. , Johanning, E. J. , David, J. R. , & Pool, J. E. (2016). Life history evolution and cellular mechanisms associated with increased size in high‐altitude Drosophila. Ecology and Evolution, 6(16), 5893–5906. 27547363 10.1002/ece3.2327 PMC4983600

[acel13576-bib-0031] Langley‐Evans, S. C. (2015). Nutrition in early life and the programming of adult disease: A review. Journal of Human Nutrition and Dietetics, 28, 1–14. 10.1111/jhn.12212 24479490

[acel13576-bib-0032] Layalle, S. , Arquier, N. , & Léopold, P. (2008). The TOR pathway couples nutrition and developmental timing in Drosophila. Developmental Cell, 15(4), 568–577. 10.1016/j.devcel.2008.08.003 18854141

[acel13576-bib-0033] Lee, G. D. , Wilson, M. A. , Zhu, M. , Wolkow, C. A. , De Cabo, R. , Ingram, D. K. , & Zou, S. (2006). Dietary deprivation extends lifespan in *Caenorhabditis* *elegans* . Aging Cell, 5(6), 515–524. 10.1111/j.1474-9726.2006.00241.x 17096674 PMC2546582

[acel13576-bib-0034] Liu, S. , Li, K. , Gao, Y. , Liu, X. , Chen, W. , Ge, W. , Feng, Q. , Palli, S. R. , & Li, S. (2018). Antagonistic actions of juvenile hormone and 20‐hydroxyecdysone within the ring gland determine developmental transitions in *Drosophila* . Proceedings of the National Academy of Sciences of the United States of America, 115(1), 139–144. 29255055 10.1073/pnas.1716897115 PMC5776822

[acel13576-bib-0035] Martins, V. J. , Toledo Florêncio, T. M. , Grillo, L. P. , Do Carmo, P. , Franco, M. , Martins, P. A. , Clemente, A. P. G. , Santos, C. D. , Vieira, M. D. F. A. , & Sawaya, A. L. (2011). Long‐lasting effects of undernutrition. International Journal of Environmental Research and Public Health, 8(6), 1817–1846. 10.3390/ijerph8061817 21776204 PMC3137999

[acel13576-bib-0036] May, C. M. , Doroszuk, A. , & Zwaan, B. J. (2015). The effect of developmental nutrition on life span and fecundity depends on the adult reproductive environment in *Drosophila melanogaster* . Ecology and Evolution, 5(6), 1156–1168. 25859322 10.1002/ece3.1389 PMC4377260

[acel13576-bib-0037] Mirth, C. K. , Tang, H. Y. , Makohon‐Moore, S. C. , Salhadar, S. , Gokhale, R. H. , Warner, R. D. , Koyama, T. , Riddiford, L. M. , & Shingleton, A. W. (2014). Juvenile hormone regulates body size and perturbs insulin signaling in *Drosophila* . Proceedings of the National Academy of Sciences of the United States of America, 111(19), 7018–7023. 10.1073/pnas.1313058111 24778227 PMC4024895

[acel13576-bib-0038] Mostoslavsky, R. , Chua, K. F. , Lombard, D. B. , Pang, W. W. , Fischer, M. R. , Gellon, L. , Liu, P. , Mostoslavsky, G. , Franco, S. , Murphy, M. M. , Mills, K. D. , Patel, P. , Hsu, J. T. , Hong, A. L. , Ford, E. , Cheng, H.‐L. , Kennedy, C. , Nunez, N. , Bronson, R. , … Alt, F. W. (2006). Genomic instability and aging‐like phenotype in the absence of mammalian *SIRT6* . Cell, 124(2), 315–329. 10.1016/j.cell.2005.11.044 16439206

[acel13576-bib-0039] Muggeo, V. M. (2003). Estimating regression models with unknown break‐points. Statistics in Medicine, 22(19), 3055–3071. 10.1002/sim.1545 12973787

[acel13576-bib-0040] Murphy, C. T. , & Hu, P. J. (2018). Insulin/insulin‐like growth factor signaling in *C*. *elegans* . WormBook: The Online Review of C. elegans Biology [Internet], 1–43. 10.1895/wormbook.1.164.1 PMC4780952 24395814

[acel13576-bib-0041] Palgunow, D. , Klapper, M. , & Döring, F. (2012). Dietary restriction during development enlarges intestinal and hypodermal lipid droplets in *Caenorhabditis* *elegans* . PLoS One, 7(11), e46198. 10.1371/journal.pone.0046198 23185233 PMC3502458

[acel13576-bib-0042] Parik, S. , Tewary, S. , Ayyub, C. , & Kolthur‐Seetharam, U. (2018). Loss of mitochondrial SIRT4 shortens lifespan and leads to a decline in physical activity. Journal of Biosciences, 43(2), 243–247. 10.1007/s12038-018-9754-5 29872013

[acel13576-bib-0043] Parlee, S. D. , & MacDougald, O. A. (2014). Maternal nutrition and risk of obesity in offspring: the Trojan horse of developmental plasticity. Biochimica Et Biophysica Acta (BBA) – Molecular Basis of Disease, 1842(3), 495–506. 10.1016/j.bbadis.2013.07.007 23871838 PMC3855628

[acel13576-bib-0044] Radimerski, T. , Montagne, J. , Hemmings‐Mieszczak, M. , & Thomas, G. (2002). Lethality of *Drosophila* lacking TSC tumor suppressor function rescued by reducing dS6K signaling. Genes & Development, 16(20), 2627–2632. 12381661 10.1101/gad.239102 PMC187466

[acel13576-bib-0045] Rehman, N. , & Varghese, J. (2021). Larval nutrition influences adult fat stores and starvation resistance in *Drosophila* . PLoS One, 16(2), e0247175. 10.1371/journal.pone.0247175 33606785 PMC7895371

[acel13576-bib-0046] Reis, T. (2016). Effects of synthetic diets enriched in specific nutrients on *Drosophila* development, body fat, and lifespan. PLoS One, 11(1), e0146758. 10.1371/journal.pone.0146758 26741692 PMC4704830

[acel13576-bib-0047] Roichman, A. , Elhanati, S. , Aon, M. A. , Abramovich, I. , Di Francesco, A. , Shahar, Y. , Avivi, M. Y. , Shurgi, M. , Rubinstein, A. , Wiesner, Y. , Shuchami, A. , Petrover, Z. , Lebenthal‐Loinger, I. , Yaron, O. , Lyashkov, A. , Ubaida‐Mohien, C. , Kanfi, Y. , Lerrer, B. , Fernández‐Marcos, P. J. , … Cohen, H. Y. (2021). Restoration of energy homeostasis by *SIRT6* extends healthy lifespan. Nature Communications, 12(1), 1–18. 10.1038/s41467-021-23545-7 PMC8163764 34050173

[acel13576-bib-0048] Sejour, R. , Sanguino, R. A. , Mikolajczak, M. , Ahmadi, W. , & Villa‐Cuesta, E. (2020). Sirt4 modulates oxidative metabolism and sensitivity to rapamycin through species‐dependent phenotypes in *Drosophila* mtDNA haplotypes. G3: Genes, Genomes, Genetics, 10(5), 1599–1612. 32152006 10.1534/g3.120.401174 PMC7202034

[acel13576-bib-0049] Shingleton, A. W. , Mirth, C. K. , & Bates, P. W. (2008). Developmental model of static allometry in holometabolous insects. Proceedings of the Royal Society B: Biological Sciences, 275(1645), 1875–1885. 10.1098/rspb.2008.0227 PMC2593922 18460425

[acel13576-bib-0050] Simon, A. F. , Shih, C. , Mack, A. , & Benzer, S. (2003). Steroid control of longevity in *Drosophila melanogaster* . Science, 299(5611), 1407–1410. 12610309 10.1126/science.1080539

[acel13576-bib-0051] Sundaresan, N. R. , Vasudevan, P. , Zhong, L. , Kim, G. , Samant, S. , Parekh, V. , Pillai, V. B. , Ravindra, P. V. , Gupta, M. , Jeevanandam, V. , & Cunningham, J. M. (2012). The sirtuin SIRT6 blocks IGF‐Akt signalling and development of cardiac hypertrophy by targeting c‐Jun. Nature Medicine, 18(11), 1643–1650. 10.1038/nm.2961 PMC4401084 23086477

[acel13576-bib-0052] Tasselli, L. , Zheng, W. , & Chua, K. F. (2017). *SIRT6*: novel mechanisms and links to aging and disease. Trends in Endocrinology & Metabolism, 28(3), 168–185. 10.1016/j.tem.2016.10.002 27836583 PMC5326594

[acel13576-bib-0053] Tu, M. P. , & Tatar, M. (2003). Juvenile diet restriction and the aging and reproduction of adult *Drosophila melanogaster* . Aging Cell, 2(6), 327–333. 10.1046/j.1474-9728.2003.00064.x 14677635

[acel13576-bib-0060] Van Voorhies, W. A. , Khazaeli, A. A. , & Curtsinger, J. W. (2004). Lack of correlation between body mass and metabolic rate in Drosophila melanogaster. Journal of insect physiology, 50(5), 445–453. 15121458 10.1016/j.jinsphys.2004.03.002

[acel13576-bib-0054] Watkins, A. J. , & Sinclair, K. D. (2014). Paternal low protein diet affects adult offspring cardiovascular and metabolic function in mice. American Journal of Physiology – Heart and Circulatory Physiology, 306(10), H1444–H1452. 10.1152/ajpheart.00981.2013 24658019

[acel13576-bib-0055] Wood, J. G. , Schwer, B. , Wickremesinghe, P. C. , Hartnett, D. A. , Burhenn, L. , Garcia, M. , Li, M. , Verdin, E. , & Helfand, S. L. (2018). Sirt4 is a mitochondrial regulator of metabolism and lifespan in Drosophila melanogaster. Proceedings of the National Academy of Sciences of the United States of America, 115(7), 1564–1569. 29378963 10.1073/pnas.1720673115 PMC5816209

[acel13576-bib-0056] Yamanaka, N. , Rewitz, K. F. , & O'Connor, M. B. (2013). Ecdysone control of developmental transitions: Lessons from *Drosophila* research. Annual Review of Entomology, 58, 497–516. 10.1146/annurev-ento-120811-153608 PMC4060523 23072462

[acel13576-bib-0057] Zheng, J. , Mutcherson, R. , & Helfand, S. L. (2005). Calorie restriction delays lipid oxidative damage in *Drosophila melanogaster* . Aging Cell, 4(4), 209–216. 10.1111/j.1474-9726.2005.00159.x 16026335

