# Supplementary material for: Drosophila Sirtuin 6 mediates developmental diet‐dependent programming of adult physiology and survival
Source: Aging Cell. 2022 Mar 2;21(3):e13576. doi: 10.1111/acel.13576 (PMC8920434; doi:10.1111/acel.13576)
Supplement: Supplementary file 1 — Supplementary Material [file ACEL-21-e13576-s001.pdf]

Supporting Information

Experimental Procedures:

Primer Sequences used in this paper

Gene Name	Forward Primer	Reverse Primer
rp49	AGTATCTGATGCCCAACATC	ACAATCTCCTTGCGCTTC
Sirt6	TCGGGACACGTTGTCCTCCAC	TCTGGCTTCATCGAAGGAAACAT
Spargel	CATTACCATGGCCAAGAACAAGGTGC	CGGGTGGAGTTTGTTGCTGAGTGGC
TFAM	CCAGCATCAGCAACACTCC	GGCATCGGACCAGTTCTTAG
Delg	CAAGTTTAAGCGAGAACAAATACGCCTG	TACATTCTTTTAGCAGCTGGAGGTG
Cox4	GGCGTTTCACTCCTCTTCTG	CTGTTGTTCTCGTAGTCCCACTTG
EiP74EF	GCGAATACTGTCCGCGTTTC	GATGTCCTTGGGCACATCCA
Eip75B	GCGGTCCAGAATCAGCAG	GAGGATGTGGAGGAGGATGA
BR-C	AACACCCGGTCATACTGCTG	ATGGCTGTGTGTGTCCTCTG
EcR	ATTTGGTGCTTGTGCCGAAC	AAGGATCATAGCTTCCGGCG
Ultraspiracle	CAGTATCCGCCTAACCATCC	TTCCTCTGCCGCTTGTCTAT
Kr-h1	CCGAATACGACATAACAGCC	CCCGATTTCCGTGAATATGT
dilp2	AGCAAGCCTTTGTCCTTCATCTC	ACACCATACTCAGCACCTCGTTG
dilp5	GAGGCACCTTGGGCCTATTC	CATCTGGTGAGATTCGGAGC
Sir2	TATATCCCGGCGAGTTTCAG	GAGGCCGTTGAAAAGGAGCCG
Sirt4	TGTCCAAACCGAATGTTTTGGTTC	GTTCCTGGCCCAGTACCGCTT
Sirt7	ACGTGGCGCAGTCTTACATAA	TTCGATCTTCCGTCGTGCG
InR	ACGATGTAGAGGGCAGTGTA	TACCACGAAGATAGCGTAGC
GSK	ATATACAGATCTTTTGTTTGGCAA	AGGAGGAAGTTCTTGGACGA
AKT	TCTCCGTACAGGGAACGACT	TGGACTTGAGCACACGACTC
Raptor	GCGACTGTTTGACAAGCGTT	CGTCCCACTCGTACACAACA
Rheb	CGACGTAATGGGCAAGAAAT	CAAGACAACCGCTCTTCTCC
4eBP	CCAGGAAGGTTGTCATCTCG	GTTGGACGGCGGAGTTTG
GLUT1	GGATTGGCCTCAGGTCTCAC	GCGAAAATGGATACCGCCAC
HexA	GTAACGTATGTGCAGGATCT	ATCATAATGTGCTGCGGTAT
PEPCK	CATTGCGTGGATGAAGTTTG	ATGCCCTCCCAGAACACAC
FBPase	TCATCAACATGCTGAAGTCA	CGATGTATTTGCCCTGTTTC
LDH	GTGTGACATCCGTGGTCAAG	CTACGATCCGTGGCATCTTT
PFK	CTGCAGCAGGATGTCTACCA	GTCGATGTTCGCCTTGATCT
PDK1	GGTCGTTACATAGGCGAGGG	AAAGAGAACGCTGGTCCTGG
Tpi	GACTGGAAGAACGTGGTGGT	CGTTGATGATGTCCACGAAC
Mondo	GCGGCGTTACAACATAAAGA	CTCCATGCGCAAAGCTTCAA
HIF1a	CCAAAGGAGAAAAGAAGGAAC	GAATCTTGAGGAAAGCGATG
SREBP	TGGCTTCTACCAAGTGCCAG	CAAGAGCTGTTGCGTTGGAC
Brummer	TCCCGAGTTTCTGTCCAAGT	GCGTCCTTTCTGTGCTTCTT
Lipase	GATTTGGGCATAGAGATGTG	CTCATAGGGATTACGGAGAG
Trehalase	TATCGGCTTCGGTTATGG	CGGGAGTGTTCAGGTTGTTT
ACC	CATTGTTAGTGTGTGGCTGTGA	CTTATCTCTGGCTCTGAACG
DAG	ATCTCCCTTAGCCTGTTTAG	TAACGAAGGTCGCCATAAG
MCAD	CAACCACATTCCTGCTGA	TCTTCTGTTCCTTGTTACCG

FAS	CGGAGAAGAGTTACATCCTG	CAATCACCACCTTTACGC
CptI	AGGAACTGCAGCCTATCATGG	GGAGTTGCTTTGCCCTTCAG
SCD1	TTTCTTCGCCAAGATCGGCT	CAATCTCCTCCTTGGGCTGG
DGAT	ACTGCTCTGCATTGGAGGTC	ATGTCCTTCGCCTTCGTTGT
Thiolase	GAAGAACAGCCAGAATATCG	CACGATGTAGTCGATGAGC
MnSOD	AGGCCAAGTCGAAGAGCGA	ACGGTCAGCGTGGTCAGCTC
Catalase	GACGATGTACATTCAGGTCA	CACCTCAGCAAAGTAGTTCT
Thioredoxin1	GGTGCGTTCTATTGTGCT	ACGGTGTCGTAAACATCCT
GSTD2q	TACGGCAAGGATGACTATCT	CAGGAAGGTGTCGAGAAAT

Supplementary Figures and Legends:

Supplementary Figure 1:

(A) Protein sequence alignment of *SIRT6* from *Drosophila,* mouse and human (uniprot.org) using ClustalW, indicating conserved active site residues, NAD+ binding and catalytic domain. (B) Schematic representation of chromosomal position indicating site of CRISPR mediated deletion of *Sirt6* and insertion of knock-in cassette containing STOP codons and RFP. (C) Genome sequence pre- and post CRISPR mediation deletion-insertion, as indicated. Primers used for confirmation are highlighted in yellow. (D) PCR based confirmation of STOP-RFP cassette insertion in *Sirt6*$^{-/-bck-L1/L2}$ flies. L1 and L2 indicate two independently generated fly lines (male-M and female-F) were confirmed for *Sirt6* deletion. (E) Quantitative PCR showing absence of *Sirt6* mRNA in CRISPR mutants of *Sirt6* relative to *w*^{1118} controls. (F) Larval weights at 24, 48 and 72 hours post synchronised egg laying of *w*^{1118} and *Sirt6*$^{-/-bck-L1}$ flies (N=3, n=30-40 per genotype). (G) Quantitative PCR to confirm over-expression of *Sirt6* mRNA from *Sirt6*^{OE} transgenic flies (*actingal4>Sirt6*^{OE}) relative to control (*actingal4>w*^{1118}). (H) Larval body size at 24, 48 and 72 hours post synchronised egg laying in *actingal4>w*^{1118} and transgenic *Sirt6* over-expressing flies. (I) Percentage pupation in control and *Sirt6* over-expressing transgenic flies (N=3, n=150-200). (J) Body weight in control and *Sirt6* over-expressing transgenic flies (N=3, n=20-25 per genotype). (K-L) Relative wing dimensions (K) and wing aspect ratio (L) in control and *Sirt6* over-expressing transgenic flies (N=3, n=20-25). (M) Quantitative PCR showing *UAS-Sirt6 (Sirt6*^{OE}*)* mediated rescue of *Sirt6* mRNA expression in *Sirt6*$^{-/-bck-1}$ flies. Relative changes in *Sirt6* expression are computed with respect to control *UAS-Sirt6 (Sirt6*^{OE}*)* flies*.* All data presented is mean ± s.e.m. (N=3, n=3). Asterisk depicts p values (*$p<0.05$, **$p<0.01$ and ***$p<0.001$) as observed by Student's t-test.

Supplementary Figure 2:

(A) Relative change in *Sirt6* mRNA expression (black dashed line) and NAD+ levels (green) in *w*^{1118} larvae during development, as indicated. (B) Relative expression of genes in *Sirt6*$^{-/-bck-L1}$ larvae compared to *w*^{1118} (black dashed line) during the time course of larval development. Asterisk depicts comparison with *w*^{1118} at 24 hours and hashtags depict comparison of the *Sirt6*$^{-/-bck-L1}$ to *w*^{1118}*,* at the respective time points, as indicated. All data presented is mean ± s.e.m. (N=3, n=3). (C) Relative change in expression of *dilp2* and *dilp5* in transgenic rescue of

Sirt6 larvae at 24, 48 and 72 hours post egg laying (N=3, n=3 with 10-20 larvae per n). Asterisk and hashtags depict p values (*, #p<0.05, **, ##p<0.01 and ***, ###p<0.001) as observed by Student's t-test and Two-way ANOVA, as applicable.

Supplementary Figure 3:

(A) Starvation survival in 3-5 day old *w*^{1118} and *Sirt6*$^{-/-bck-L1/L2}$ flies (N=3, n=6 with 10 flies per n). (B) Oxidative stress survival on 20mM Paraquat in *w*^{1118} (control) and *Sirt6*$^{-/-bck-L1}$ flies (N=3, n=6 with 10 flies per n). (C) Relative mRNA levels of genes involved in mitochondrial biogenesis *w*^{1118} and *Sirt6*$^{-/-bck-L1/L2}$ flies (N=3, n=3 with 8 flies per n). (D) Representative blots and quantification (right) for change in levels of mitochondrial proteins in *w*^{1118} and *Sirt6*$^{-/-bck-L1/L2}$ flies (N=3, n=3 with 8 flies per n). (E) Mitochondrial DNA content (normalised to nuclear DNA) in 35-37 day old *w*^{1118} (control) and *Sirt6*$^{-/-bck-L1/L2}$ flies (N=2, n=3 with 30 flies per n). (F) Mitochondrial DNA content (normalised to nuclear DNA) in transgenic rescue of *Sirt6* in 35-37 days old flies. (G) Average number of eggs laid per fly over a period of 20 days from a heterogeneous cross, parents as indicted (N=5, n=10). All data presented is mean ± s.e.m. Asterisk depict p values (*,$^{\#}$p<0.05, **,$^{\#\#}$p<0.01 and ***,$^{\#\#\#}$p<0.001) as observed by Student's t-test and Two-way ANOVA, as applicable.

Supplementary Figure 4:

(A-B) Weight gain and pupation onset (marked in black) in *w*^{1118} larvae reared on differential concentration of yeast (A) and glucose (B) diets, as indicated (N=3, n=20-25). (C) Weight gain and pupation onset (marked in black) in *w*^{1118} and *Sirt6*$^{-/-bck-L1}$ larvae reared on differential concentration of glucose diets, as indicated (N=3, n=20-25). (D) Body weight measurement in *w*^{1118} and *Sirt6*$^{-/-bck-L1}$ 3-5 day old flies (N=3, n=20-25 per genotype), reared on differential concentration of glucose diets, as indicated. (E) Median survival under starvation in *w*^{1118} and *Sirt6*$^{-/-bck-L1}$ flies reared on differential concentrations of yeast and glucose diets, from three independent experiments (N=3, n=8 with 10 flies per n). (F) Median survival under oxidative stress in *w*^{1118} and *Sirt6*$^{-/-bck-L1}$ flies reared on differential concentrations of yeast and glucose diets, from three independent experiments (N=3, n=8 with 10 flies per n). All data presented is mean ± s.e.m. Student's t-test and Two-way ANOVA were used to analyse statistical significance of the data (*p<0.05, **p<0.01 and ***p<0.001).

Supplementary Figure 5:

(A-B) Life spans of w^{1118} (A) and *Sirt6*$^{-/-bck-1}$ (B) flies on normal diet, which were reared on differential yeast diets during larval development (n = 10 with 10 flies per n). (C-D) Life spans of w^{1118} (C) and *Sirt6*$^{-/-bck-1}$ (D) flies on normal diet, which were reared on differential glucose diets during larval development (n = 10 with 10 flies per n). (E) Median life spans of w^{1118} and *Sirt6*$^{-/-bck-1}$ reared on differential yeast and glucose diets (N=3, n = 10 with 10 flies per n). Log-rank (Mantel-Cox) test was used to plot survival curves and statistical analysis. Asterisks indicate comparison with w^{1118} grown on ND and hashtag indicates comparison among w^{1118} and *Sirt6*$^{-/-bck-1}$ for the particular diet. All data presented is mean ± s.e.m. Two-way ANOVA was used to analyse statistical significance of the data (*, $^{\#}p<0.05$, **, $^{\#\#}p<0.01$ and ***, $^{\#\#\#}p<0.001$).

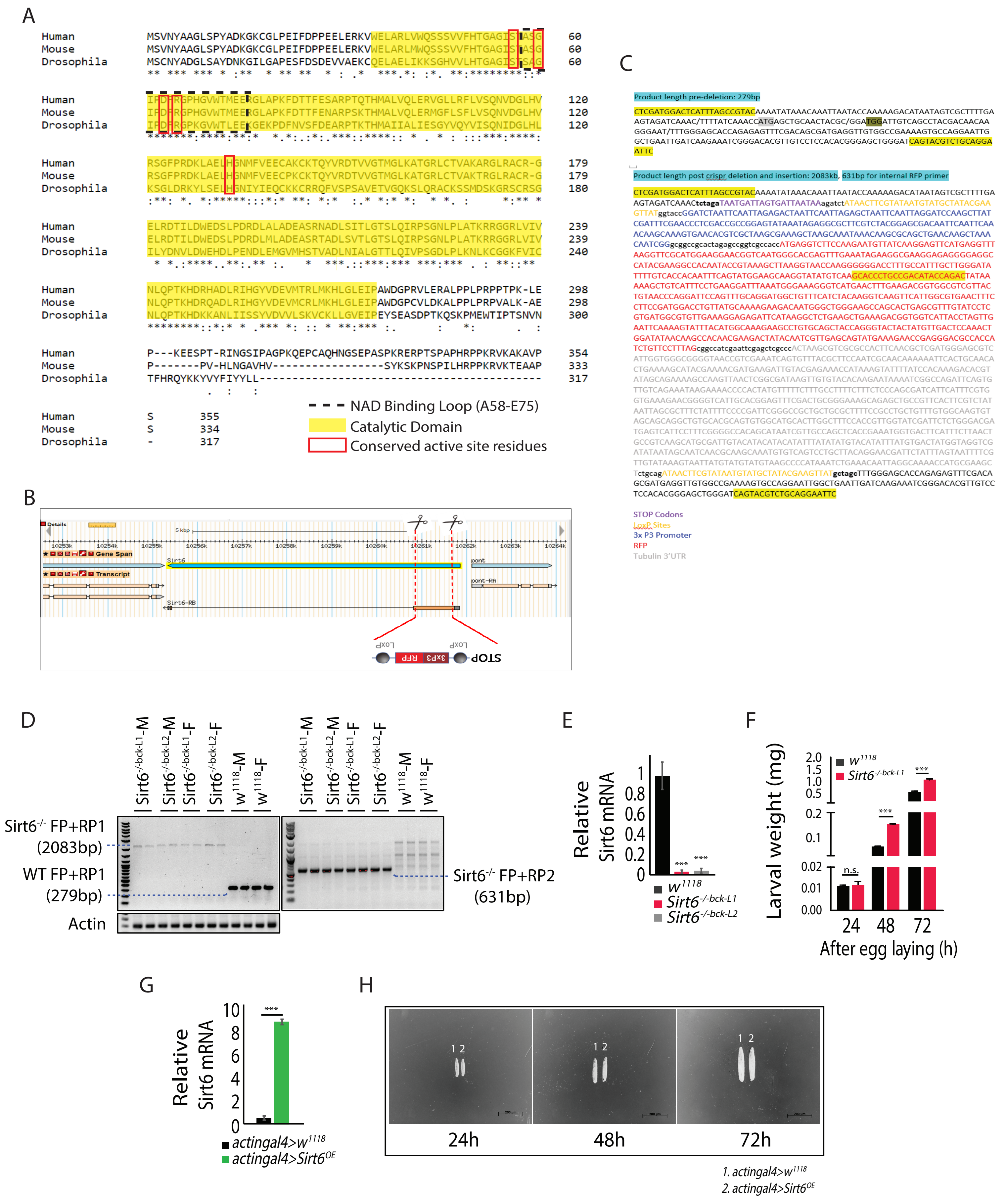

Supplementary figure 1

Contd.

Contd.

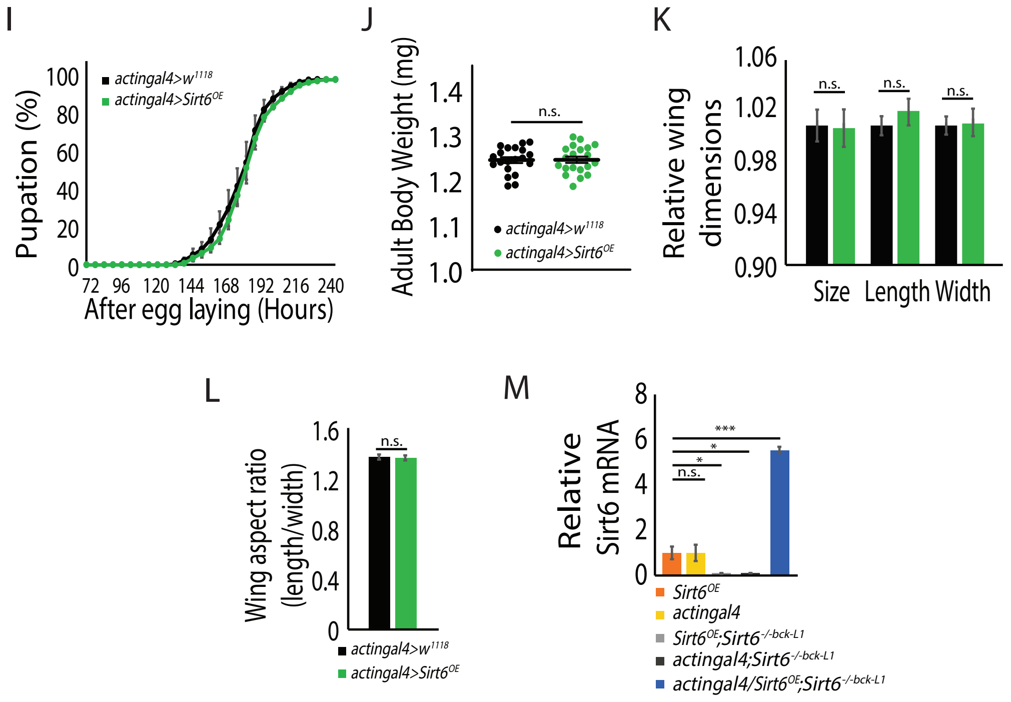

Supplementary figure 1

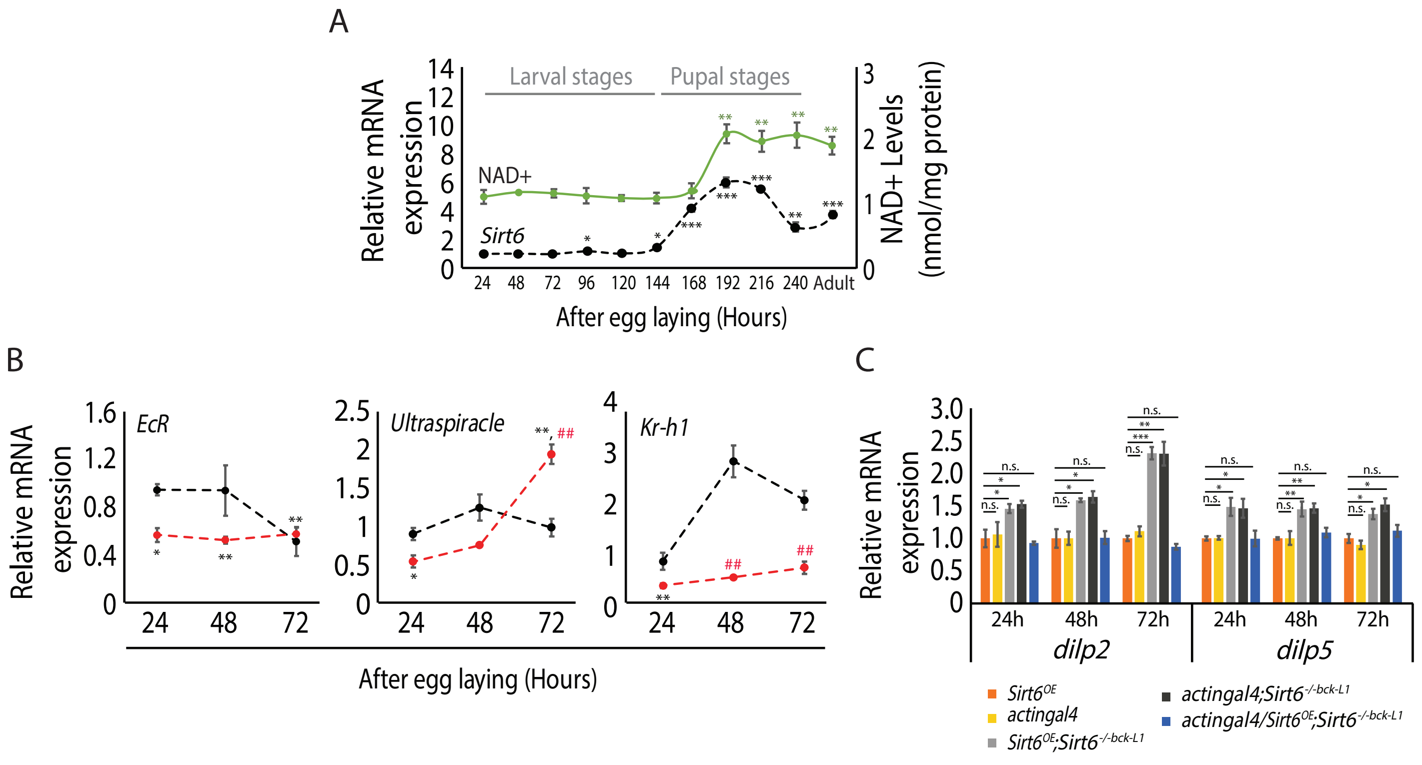

Supplementary figure 2

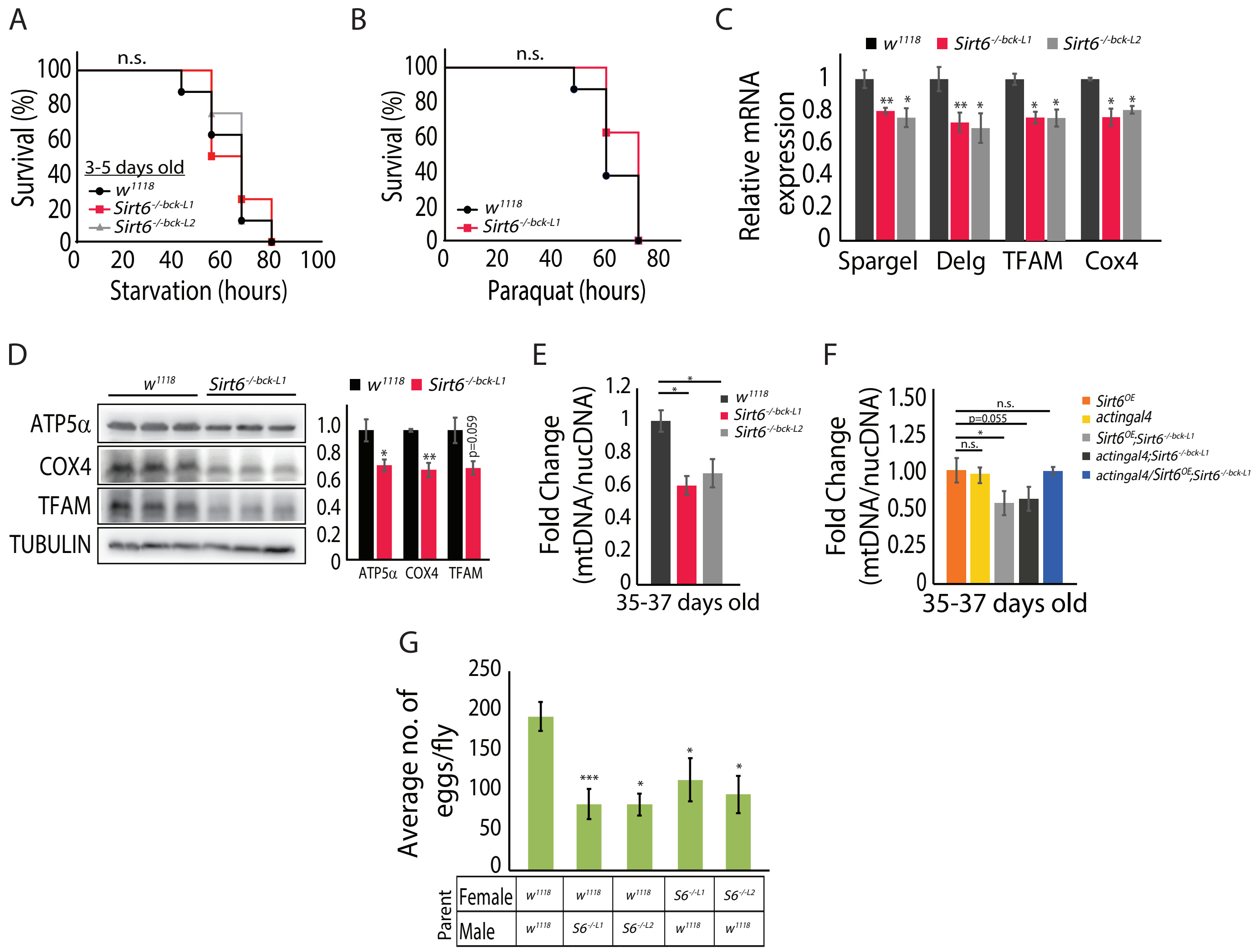

Supplementary figure 3

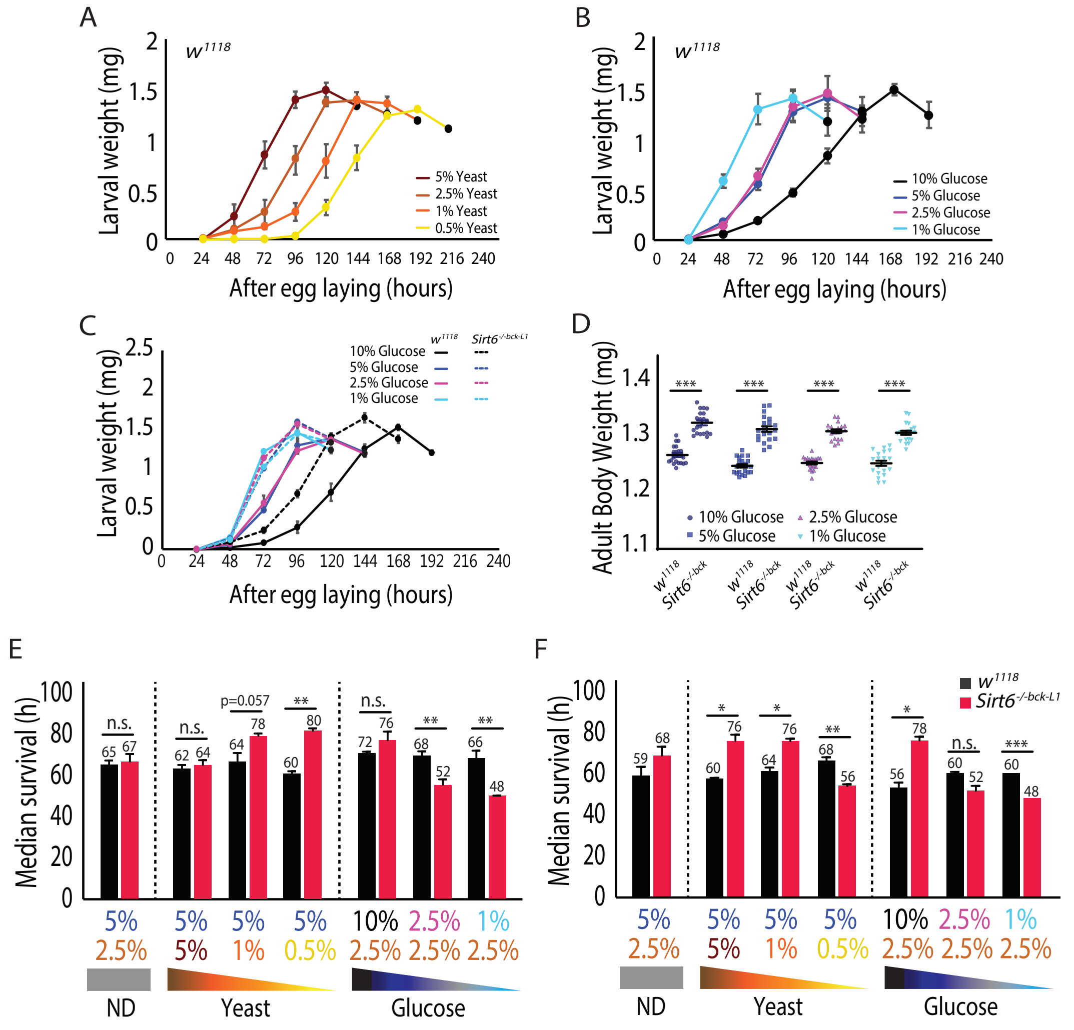

Supplementary figure 4

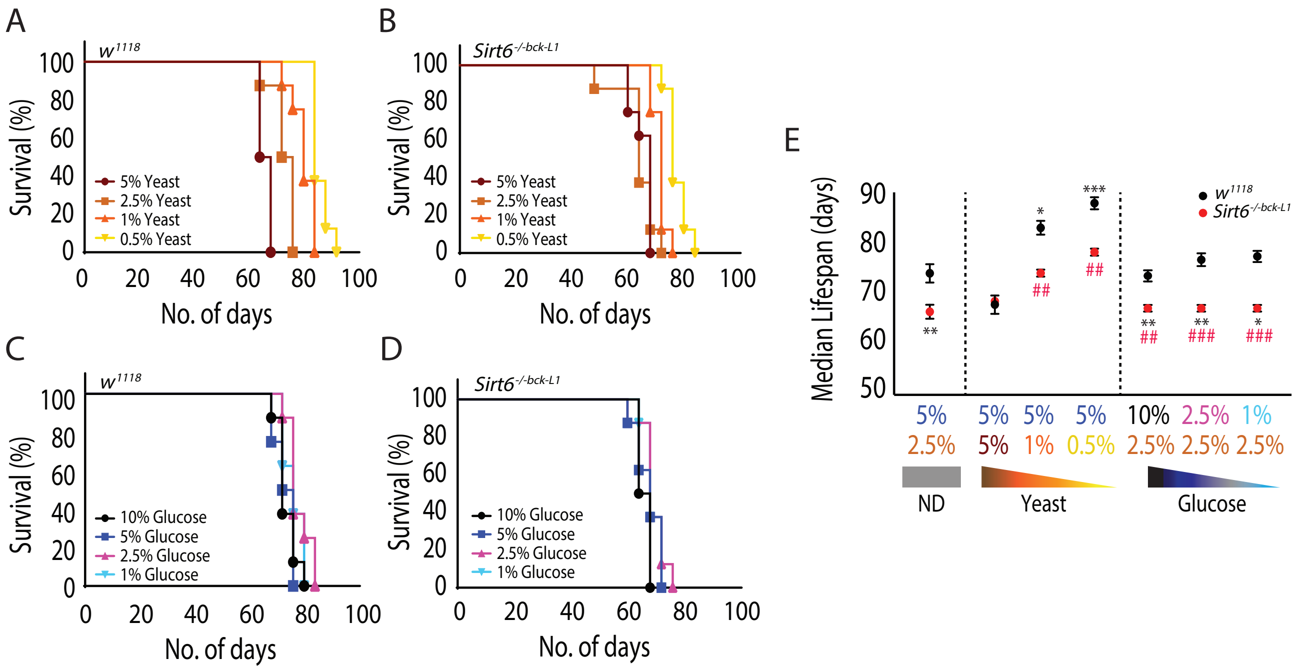

Supplementary Figure 5
